# Connecting thiamine availability to the microbial community composition in Chinook salmon spawning habitats of the Sacramento River basin

**DOI:** 10.1128/aem.01760-23

**Published:** 2023-12-12

**Authors:** Christopher P. Suffridge, Kelly C. Shannon, H. Matthews, R. C. Johnson, C. Jeffres, N. Mantua, A. E. Ward, E. Holmes, J. Kindopp, M. Aidoo, F. S. Colwell

**Affiliations:** 1 Department of Microbiology, Oregon State University, Corvallis, Oregon, USA; 2 Fisheries Ecology Division, NOAA Fisheries, Southwest Fisheries Science Center, Santa Cruz, California, USA; 3 University of California, Center for Watershed Sciences, Davis, California, USA; 4 California Department of Water Resources, West Sacramento, California, USA; 5 California Department of Water Resources, Division of Integrated Science and Engineering, Oroville, California, USA; 6 Bronx Community College, Bronx, New York, USA; 7 College of Earth, Ocean, and Atmospheric Sciences, Oregon State University, Corvallis, Oregon, USA; University of Michigan, Ann Arbor, Michigan, USA

**Keywords:** thiamine, fish health, aquatic microbiology, salmonids

## Abstract

Thiamine deficiency complex (TDC) is a major emerging threat to global populations of culturally and economically important populations of salmonids. Salmonid eggs and embryos can assimilate exogenous thiamine, and evidence suggests that microbial communities in benthic environments can produce substantial amounts of thiamine. We therefore hypothesize that natural dissolved pools of thiamine exist in the surface water and hyporheic zones of riverine habitats where salmonids with TDC migrate, spawn, and begin their lives. To examine the relationship between dissolved thiamine-related compounds (dTRCs) and their microbial source, we determined the concentrations of these metabolites and the compositions of microbial communities in surface and hyporheic waters of the Sacramento River, California and its tributaries. Here we determine that all dTRCs are present in femto-picomolar concentrations in a range of critically important salmon spawning habitats. We observed that thiamine concentrations in the Sacramento River system are orders of magnitude lower than those of marine waters, indicating substantial differences in thiamine cycling between these two environments. Our data suggest that the hyporheic zone is likely the source of thiamine to the overlying surface water. Temporal variations in dTRC concentrations were observed where the highest concentrations existed when Chinook salmon were actively spawning. Significant correlations were seen between the richness of microbial taxa and dTRC concentrations, particularly in the hyporheic zone, which would influence the conditions where embryonic salmon incubate. Together, these results indicate a connection between microbial communities in freshwater habitats and the availability of thiamine to spawning TDC-impacted California Central Valley Chinook salmon.

**IMPORTANCE:**

Pacific salmon are keystone species with considerable economic importance and immeasurable cultural significance to Pacific Northwest indigenous peoples. Thiamine deficiency complex has recently been diagnosed as an emerging threat to the health and stability of multiple populations of salmonids ranging from California to Alaska. Microbial biosynthesis is the major source of thiamine in marine and aquatic environments. Despite this importance, the concentrations of thiamine and the identities of the microbial communities that cycle it are largely unknown. Here we investigate microbial communities and their relationship to thiamine in Chinook salmon spawning habitats in California’s Sacramento River system to gain an understanding of how thiamine availability impacts salmonids suffering from thiamine deficiency complex.

## INTRODUCTION

Thiamine deficiency complex (TDC) is an emerging threat to global populations of salmonid fish (1–3). TDC is caused by chronic low systemic concentrations of thiamine (vitamin B_1_), resulting in early life-stage mortality (1, 4). The occurrence of thiamine deficiency has been globally documented in habitats including the Baltic Sea, the New York Finger Lakes, the Laurentian Great Lakes, the Yukon River in Alaska, the Sacramento-San Joaquin River watershed in California, and coastal rivers in Oregon (4–7). Many studies have linked TDC occurrence to salmonid consumption of planktivorous fish species containing the thiamolytic enzyme, thiaminase (1, 5, 8–15). Clupeid prey with observed thiaminase activity include anchovies, herring, sprat, and alewife, which can each contribute to the bulk of salmonid diets in various aquatic ecosystems and can contribute to TDC (1, 6, 8, 12). A second, yet not mutually exclusive, hypothesis links TDC incidence to the consumption of prey fish with high lipid and energy densities, such as sprat, which causes oxidative stress and results in thiamine depletion in predators (16). Incidences of TDC have increased globally in multitudes of animal species (2) and it is now considered a major emerging threat to the stability of many globally distributed populations of salmonids and their associated fisheries (1, 2).

**Fig 1:**
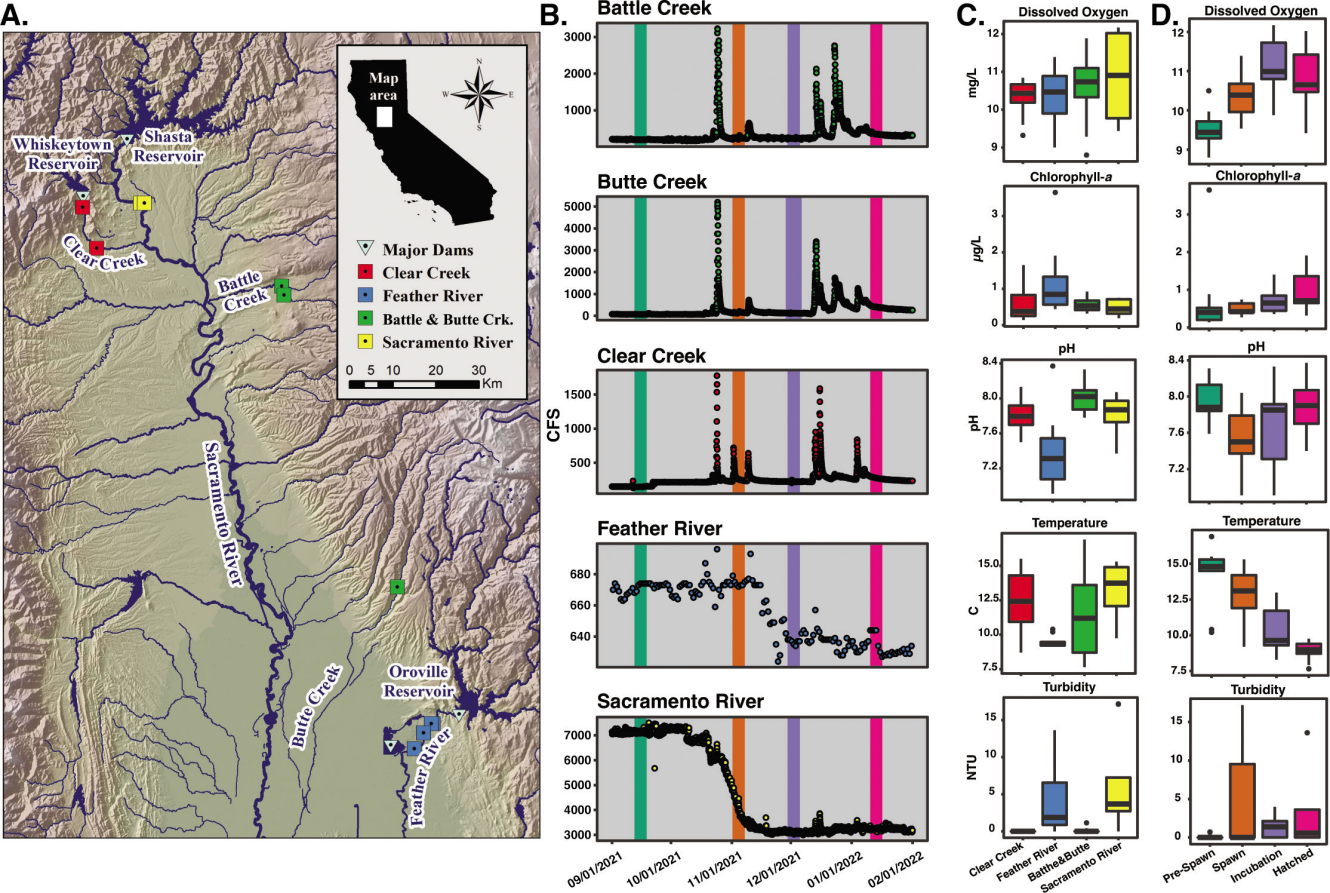
Geographic, physical, and chemical parameters of sampling sites. (A) Sample locations in the Sacramento River watershed. Sample locations are coded by color throughout the manuscript: Sacramento River (yellow), Clear Creek (red), Feather River (blue), and Battle and Butte Creeks (green). (B) Hydrological data from each stream from September 2021 to February 2022 (cubic feet per second). Colored bars represent sampling times, and these colors are used throughout the manuscript: Pre-Spawn (teal), Spawn (orange), Incubation (purple), and Hatched (pink). (C) Box plots of water chemistry data binned by the river. (D) Box plots of water chemistry data binned by sampling event.

TDC has recently been diagnosed for the first time in Chinook salmon populations spawning in the California Central Valley, including those spawning in the Sacramento River system (Fig. 1) (6). Symptoms of TDC in this system were first observed in recently hatched fry in hatcheries in 2020 (6). These juvenile fish displayed common symptoms of TDC including lethargy, abnormal swimming patterns, loss of equilibrium, and death (6). Fishery managers were able to successfully mitigate TDC in the hatchery populations by treating fry and eggs with thiamine baths and thiamine injections (6, 17). In this incidence, TDC was hypothesized to be linked to an unprecedented dominance of Northern Anchovy in the diets of these salmon (6). Anchovy populations in the California Current ecosystem historically undergo boom and bust cycles, often alternating in dominance with sardines, presumably in response to varying ocean conditions (18, 19). We theorize that microbial thiamine production in Chinook salmon spawning habitats may naturally mitigate the impacts of ocean-sourced TDC.

While post-embryonic salmonids primarily acquire thiamine through dietary intake, all life stages of salmonids (including eggs and embryos) can acquire thiamine directly from the dissolved pool in the surrounding environment *via* uptake over the gills and diffusion, respectively (1, 7, 20). The degree to which salmon could be supplemented by environmental dissolved thiamine at each life stage is unknown. It is unlikely that direct thiamine uptake from the dissolved pool represents a significant source of this vitamin once salmonids are actively feeding (regardless of developmental stage or habitat occupied) because their prey likely satisfies the majority of their metabolic thiamine demands. However, during adult salmon’s freshwater migration and spawning life stages when adults are fasting, and for eggs and embryos incubating in riverine sediments, direct uptake of exogenous thiamine may represent a major thiamine source. Past research has established that thiamine deficiency in salmonid embryos is maternally transmitted (21). Therefore, the environmental dissolved thiamine pool could mitigate the pathological effects of TDC experienced by spawning female salmonids.

**Fig 2:**
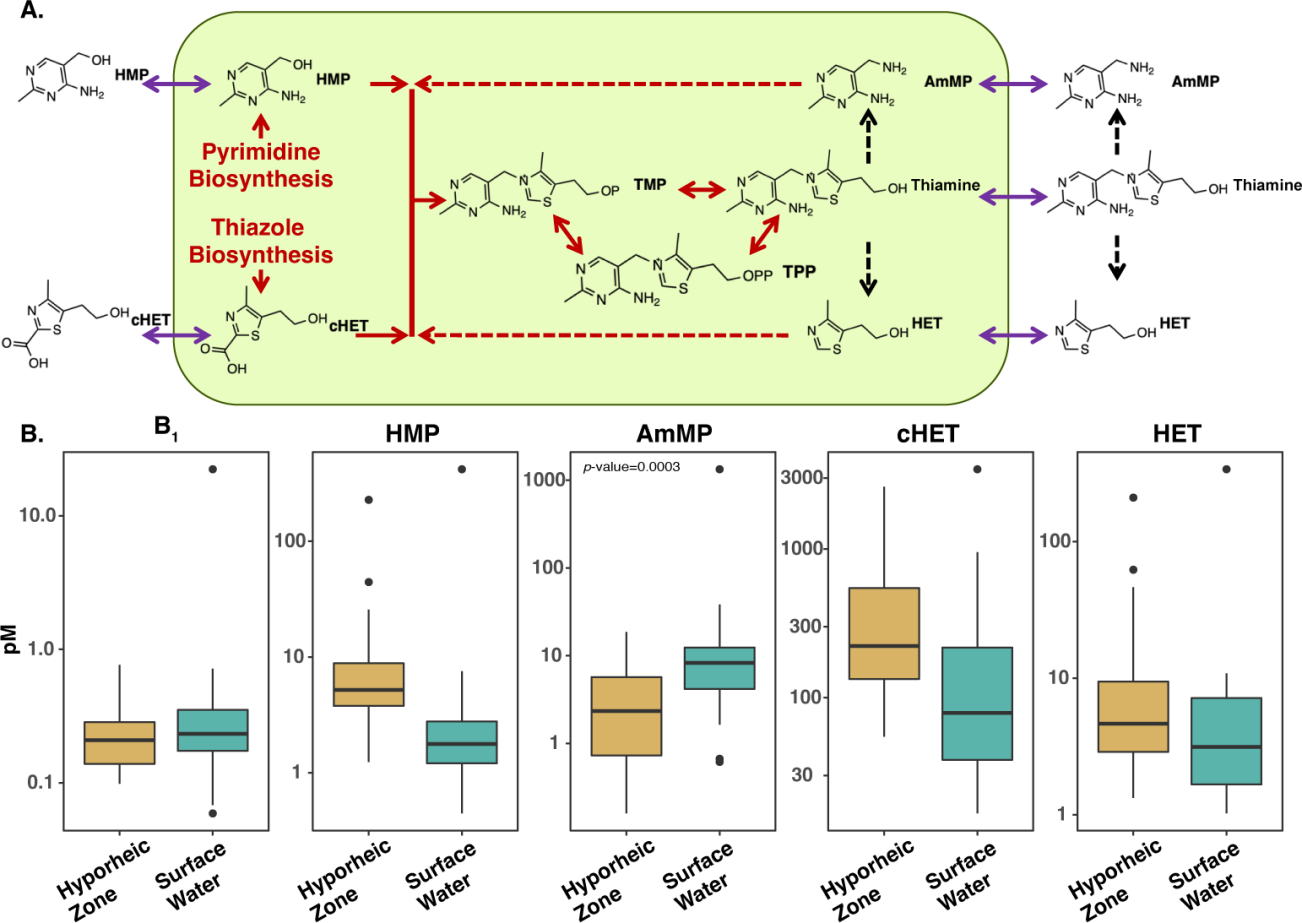
dTRCs within the Sacramento River watershed. (A) Summary of microbial thiamine cycle pathways. The green rectangle represents a generic microbial cell. Solid red arrows represent cellular biosynthetic processes. Dashed red arrows represent cellular salvage pathways. Dashed black arrows represent degradation processes. Purple double-headed arrows represent cellular excretion and uptake processes. In some cases, arrows represent multiple enzymatic and/or abiotic processes. (B) Distribution of dTRCs within the Sacramento River watershed. Boxplots compare concentrations (picomolar; pM) of all river SW (blue) and HZ (tan) samples of each dTRC. Significant differences between HZ and SW means were examined with paired *t*-tests for all dTRCs but were only identified for AmMP.

Vitamin B_1_ is an essential coenzyme universally required by all domains of life; yet, it is mainly biosynthesized by a relatively small subset of microorganisms, and most microbes are auxotrophs (obligately required) for the vitamin (22, 23). Vitamin B_1_ functions as a cofactor for roughly 2% of all characterized enzymes that require a cofactor and is essential for both catabolic and anabolic central carbon metabolism and branched-chain amino acid synthesis (24). Thiamine is a heterocyclic molecule containing pyrimidine and thiazole moieties which are synthesized by two separate biosynthesis pathways and ligated to form thiamine (25). Multiple metabolically relevant thiamine-related compounds (TRCs) must be simultaneously analyzed to fully assess environmental microbial thiamine cycling (26). The generalized microbial thiamine cycle is visually outlined in Fig. 2; the concentration of each compound can be considered as a proxy for different metabolic processes in the thiamine cycle. The primary biosynthetic precursors to thiamine (referred to as B_1_ hereafter) are the pyrimidine compound 4-amino-5-hydroxymethyl-2-methylpyrimidine (HMP) and the thiazole compound 5-(2-hydroxyethyl)−4-methyl-1,3- thiazole-2-carboxylic acid (cHET) (25, 27). These compounds are solely produced through biosynthetic processes and can be considered proxies for active thiamine biosynthesis. It has been reported that the thiazole compound 4-methyl-5-thiazoleethanol (HET) is both a B_1_ biosynthetic precursor and degradation product (produced by both intracellular and extracellular processes) that can be utilized through a salvage pathway to complete cellular B_1_ biosynthesis (22, 25, 27–32). Similarly, cells are known to be able to utilize exogenous sources of the B_1_ pyrimidine degradation product 4-amino-5-aminomethyl-2-methylpyrimidine (AmMP) through a salvage pathway to complete B_1_ biosynthesis, and thus AmMP can be viewed as a proxy for thiamine degradation (32).

The concentrations of dissolved TRCs (dTRCs) in freshwater environments are largely unknown. Less than 50 discrete freshwater dissolved B_1_ measurements exist worldwide, and concentrations are reported in the picomolar range (33–35). Measurements of B_1_ from Lake Tahoe, California and the Peconic River Estuary in New York range between 12 and 190 pM; however, these studies used bioassay and high-performance liquid chromatography (HPLC)-based methods which cannot accurately discriminate between all TRCs and are therefore likely overestimates (33, 34). Tovar-Sanchez and colleagues (2016) used a liquid chromatography-mass spectrometry (LCMS)-based approach to measure B_1_ in the Moulouya River, Africa, and observed a mean concentration of 4.5 pM (35). The concentrations of other dTRCs have never been determined in freshwater systems.

Microbial thiamine cycling has not been previously investigated in freshwater systems; however, research on marine environments indicates that while some microbes (bacteria, archaea, and eukaryotic algae) contain the complete repertoire of biosynthesis genes and produce thiamine *de novo* (prototrophs), the majority of microbes lack these pathways (auxotrophs) and must rely on exogenous dissolved thiamine precursor compounds or intact thiamine (22, 36). Specifically, many of these organisms cannot synthesize one, or both, of thiamine’s precursor moieties (e.g., HMP, cHET) and therefore must acquire these compounds (or analogous degradation products; e.g., AmMP, HET) from the exogenous dissolved pool to complete thiamine biosynthesis (22, 27–29, 32, 37). The relative abundance of thiamine prototrophic and auxotrophic algae and bacterioplankton can influence dTRC concentrations in the marine water column (26).

Marine studies have also shown that microbial dTRC production balances removal and many species of free-living bacterioplankton produce dissolved thiamine (38, 39). Furthermore, the Peconic River was shown to be a source of thiamine for the coastal ecosystem of Long Island, NY, displaying a potential connection between thiamine cycling in freshwater, estuary, and marine systems (34). Marine sediments of the Santa Monica Basin offshore of Los Angeles, California have been shown to produce substantial amounts of thiamine (40). Research in freshwater systems has shown correlative relationships between concentrations of sediment-sourced trace metals and thiamine, suggesting that the microbial inhabitants of river sediments produce thiamine in the overlying water (35). Therefore, the observed standing stock concentrations of dTRCs in any environment represent an equilibrium between thiamine production and removal.

Here we report the distribution of dTRCs coupled with microbial community compositions in freshwater systems, to assess how microbial community characteristics influence dTRC concentrations. The relationship between microbial community compositions and concentrations of dTRCs was assessed in both the surface water (SW) and hyporheic zone (HZ) of the Sacramento River basin, a freshwater, lotic environment where adult Chinook salmon spawn and their offspring rear. We hypothesize that (1) dTRC concentrations are associated with the compositions of SW and HZ microbial communities of the Sacramento River and its tributaries and (2) both dTRC concentrations and compositions of microbial communities of each sample type (HZ and SW) differ by region of the basin and time points related to the spawning of Sacramento River fall-run Chinook salmon. We seek to gain an initial understanding of how natural and anthropogenic changes can impact the structure of aquatic microbial communities and thus the availability of thiamine to higher trophic levels. Furthermore, we assess which microbial taxonomic annotations [taxa, or amplicon sequence variants (ASVs)] correlate most significantly with dTRC concentrations to help infer how the relative abundance of certain taxa can correspond to dTRC availability. Pacific salmon are keystone species (41) with considerable economic importance and immeasurable cultural significance to Indigenous peoples (42, 43). Therefore, examining the structure of microbial communities that produce and consume TRCs in rivers where TDC-impacted Chinook salmon spawn has multiple intrinsic benefits to all people.

## MATERIALS AND METHODS

### Sample collection

Samples were collected in September, November, and December of 2021 and in January of 2022. These dates were chosen to bracket key events for fall-run Sacramento River Chinook salmon, and they correspond to before spawning (Pre-Spawn), during active spawning (Spawn), during the salmon egg incubation (Incubation), and after the eggs had hatched but the embryonic fish had not yet left the gravel and started feeding exogenously (Hatched) (44). In all, 11 sites within the Sacramento River watershed were sampled at each of the four time points (Fig. 1). Sites (listed North to South) were located on the Sacramento River below Shasta and Keswick dams (SAC, SSC), Clear Creek below the Whiskeytown Dam (CCM, CCH), North Fork Battle Creek (NFB), South Fork Battle Creek (SFB), Butte Creek (BCC), and the Feather River below the Oroville Dam (FRU, FRD; FR2, FR3 only at the December sampling). Sites were chosen to capture a gradient of anthropogenic influence. Clear Creek, the Sacramento River, and the Feather River sites are all downstream of major high head dams and reservoirs that fully prevent upstream fish passage. North and South forks of Battle Creek and Butte Creek are impacted by small dams and water diversions and lack major reservoirs. Chinook salmon are known to actively spawn at all sites. The sites on the Feather River in Oroville are adjacent to a hatchery. FRU is upstream of the hatchery while sites FRD, FR2, and FR3 are downstream.

Samples were collected from both the river SW and HZ at each site. SW samples were collected directly from a depth of 0.5 m using 1 L amber HDPE bottles (Nalgene). HZ samples were collected by driving a standpipe 30 cm into the unconsolidated river gravels in areas typical of salmon spawning. The bottom 20 cm of the standpipe was perforated to allow sample collection from the HZ. A Teflon tube was then inserted into the standpipe, and the sample was extracted using a hand-powered pump. Care was taken to extract all overlying river water from the standpipe prior to sample collection. Samples were collected into 1 L amber HDPE bottles. Collected samples from both the SW and HZ were then prefiltered using a 100 µm mesh to remove all metazoans and large suspended particles. Sampling occurred in daylight hours over the course of 1 day for each sampling event. All sample collection equipment was cleaned as appropriate for trace organic compound sampling which has been described previously (45). Briefly, all bottles, tubing, and pumps were acid washed using 1 M hydrochloric acid, followed by rinses with MilliQ water and finally methanol to remove all organics. The sampling equipment was thoroughly rinsed with the sample before sample collection. Water samples were stored on ice until filtration occurred.

Concurrent with water sampling, a YSI EXO2 Multiparameter Water Quality Sonde was used to collect river metadata. Data were collected for temperature (°C), dissolved oxygen concentration (mg•L^−1^), turbidity (NTU), total dissolved solids (TDS), chlorophyll-*a* concentration (μg•L^−1^), and pH. Data for river discharge (CFS) were downloaded from the California Department of Water Resources California Data Exchange Center (https://cdec.water.ca.gov/dynamicapp/wsSensorData).

River SW and HZ water samples were processed for dTRC and microbial community analysis as has been described previously (26, 45). Briefly, gentle peristaltic filtration (30 mL•min^−1^) was used to collect all cells and particles on a 0.22-µm Sterivex filter (PES membrane, Millipore, Burlington, MA, USA). Microbial biomass on the Sterivex filter was used for 16S rRNA gene amplicon microbial community diversity analysis. After filtration, the filter was blown dry, capped, and stored at −80°C until analysis. One liter of the cell-free filtrate was collected in an acid-washed, methanol-rinsed, amber HDPE bottle for dTRC analysis. The dTRCs in the filtrate were stabilized with the addition of 1 mL of 1 M hydrochloric acid, and the filtrate was stored at −20°C until analysis. Samples were processed using a portable field laboratory to allow for processing to occur on the same day as collection. All samples were transported frozen to Oregon State University for analysis.

### dTRC analysis

dTRCs were extracted from SW and HZ samples as previously described (26, 45). Samples were thawed and adjusted to pH 6.5 using 1 M HCl or 1 M NaOH. dTRCs were extracted from the SW and HZ matrix using a solid phase extraction with Bondesil C_18_ resin (Agilent). Samples were passed over 8 mL of resin at the rate of 1 mL•min^−1^. Previous research has determined that all dTRCs were retained on the resin (26). Compounds were eluted from the column using 12 mL of LCMS grade methanol, which was then concentrated by evaporation to 250 µL using a blow-down nitrogen drier (Glass Col). This provides a six order of magnitude concentration factor between the environmental sample concentration and the concentration analyzed on the LCMS. Hydrophobic organic compounds that were co-extracted by the solid phase extraction were removed using a liquid phase extraction with 1:1 vol of chloroform. Samples were then stored at −80°C until LCMS analysis.

An LCMS method was utilized to simultaneously measure dTRC concentrations (26). Analysis was conducted using an Applied Biosystems 4000 Q-Trap triple quadrupole mass spectrometer with an ESI interface coupled to a Shimadzu LC-20AD liquid chromatograph. Applied Biosystems Analyst and ABSciex Multiquant software were used for instrument operation and sample quantification. A Poroshell 120 PFP, 3 × 150 mm 2.7 µm HPLC column (Agilent) with a Poroshell 120 PFP, 2 × 5 mm, 2.7 µm guard column (Agilent) was used for chromatographic separations. The column temperature was isocratic at 40°C. HPLC mobile phases were MS-grade water (Fisher) with 0.1% formic acid and MS-grade acetonitrile (Fisher) with 0.1% formic acid. A 15-min binary gradient was used with a flow rate of 200 µL•min^−1^ and an initial concentration of 3% acetonitrile ramping to 100% acetonitrile in 7 min and column re-equilibration at 3% acetonitrile for 6 min. A third HPLC pump with a flow rate of 100 µL•min^−1^ acetonitrile (0.1% formic acid) was connected to a mixing tee post-column to increase the ionization efficiency as most dTRCs elute from the column in the aqueous phase of the gradient. The ESI source used a spray voltage of 5,200 V and a source temperature of 450°C. Curtain gas pressure was set at 30 PSI. The mass spectrometer was run in positive ion mode. Compound-specific information including MRM parameters, column retention times, and limits of detection have been previously published (26). The sample injection volume was 10 µL, and samples were analyzed in triplicate. Samples were indiscriminately randomized prior to analysis. To compensate for matrix effects, ^13^C-labeled thiamin was used as an internal standard. LCMS analysis was conducted at the Oregon State University Mass Spectrometry Core Facility.

### DNA extraction, amplification, and sequencing preparation

Microbial DNA was extracted from Sterivex filters representing approximately 1 L of SW or HZ water per sample site. Using an autoclave-sterilized scalpel, each Sterivex filter membrane was sectioned in half (representing approximately 500 mL of sample) and placed in Qiagen DNeasy Powersoil DNA isolation kit bead-beating tubes (Qiagen; Hilden, Germany) for immediate DNA extraction and the other half for storage at −80°C. Sterivex filters in each bead-beating tube were dissolved by adding 0.1 mL of phenol chloroform in a fume hood and the remainder of the DNA extraction protocol was performed following the manufacturer’s SOP. All Sterivex extraction steps prior to phenol-chloroform addition (done in a chemical fume hood) were carried out in a laminar flow hood. Negative controls were performed in parallel by adding 0.25 mL of PCR-grade water to bead-beating tubes and carrying out all DNA extraction and PCR steps.

The V4-V5 hypervariable region of the bacterial and archaeal 16S SSU rRNA gene was PCR amplified with indexed 515F-806R 16S primers (46, 47). Two separate high-throughput sequencing runs were performed on the Feather River sample sites and the remainder of the sample sites. PCR for Feather River samples was performed in 25 µL double-well reactions containing 200 nM of forward and reverse primers, 12.5 µL of Platinum II Hot-Start PCR Master Mix (2X) (Invitrogen Waltham, Massachusetts), 1 µL of undiluted DNA template, and PCR-grade sterile water to a total volume of 25 µL. The HZ FRU Spawn time period sample was PCR amplified with a 6 µL template due to a low extraction yield, and HZ Incubation-time period FR3 sample was PCR amplified with a 1 µL 1:5-diluted sample for proper PCR amplification. The volumes of all other samples contained 1 µL of undiluted template DNA. Thermocycler conditions were performed following the PCR master mix manufacturer’s procedure and consisted of the following steps for all samples: 2 min initial denaturation at 94°C, 30–35 cycles of denaturation at 94°C for 15 s, annealing at 60°C for 15 s, and extension at 72°C for 15 s, followed 10 min at 72°C for a final non-cycled hold. Feather River samples were pooled to an equimolar concentration and sequenced at the Oregon State University Center for Quantitative Life Sciences (CQLS) for 2 × 250 bp Illumina MiSeq high-throughput sequencing in June 2022 and the remainder of samples were pooled to an equimolar concentration and sequenced at the CQLS using the same sequencing platform in October 2022.

### Bioinformatics and statistics of microbial community data

The CQLS demultiplexed all raw MiSeq reads and trimmed the majority of adaptor sequences. Final Illumina adaptor trimming and initial quality filtering were performed with Trim Galore (v0.6.7) to drop reads with Phred scores of <20. FastQC (v0.11.9) was used to generate read quality reports and MultiQC (v1.14; (48)) was used for visualization of combined reports. All further computational work was performed in RStudio (v4.1.1; (49)). DADA2 (v124.0; (50)) was used to perform final read filtering, contig creation, and read assignment of prokaryotic ASVs, following default parameters, except conducting pseudo-pooling at the denoising step to allow for better recognition of rarer ASVs in each sample based on prior information (51). The Silva (v138.1; (52)) 16S reference database was used to assign taxonomy to bacteria and archaea and the Phytoref (53) reference database was used to assign plastid taxonomy to ASVs that were identified as Chloroplast sequences by Silva alignment in DADA2. Decontam (v1.16.0; (54)) was used with default parameters to remove contaminant ASVs based on the existence of these ASVs in negative control samples.

A Phyloseq object was generated from the ASV count and taxonomy tables of bacteria, archaea, and Phytoref-assigned algae from DADA2 and by integrating an associated metadata table using the phyloseq package (v1.42.0; (55)). This Phyloseq object was then rarefied to an even depth of 8,087 reads, representing 17,733 total ASVs, and all samples were maintained. Separate Phyloseq objects were generated for total bacteria and archaea (excluding algae-assigned ASVs), HZ bacteria, archaea, and Phytoref-assigned algae, and SW bacteria, archaea, and Phytoref-assigned algae. Phyloseq was used to generate Bray-Curtis dissimilarity matrices for each Phyloseq object and vegan (v2.6–4; (56)) was used to find significant differences between groups (PERMANOVA), significant differences between group variances, and to generate vectors for NMDS. All Bray-Curtis dissimilarity NMDS plots were visualized with ggplot2 (v3.4.0; (57)). Constrained analysis of principal coordinates (CAP), alpha diversity analyses, and Kruskal-Wallis tests were performed with the Phyloseq, MicrobiotaProcess (v1.8.2; (58)), and Stats (49) packages, respectively (see supplemental methods for details).

Mantel tests are performed with transformed (Tukey’s ladder of powers), continuous dTRC concentration values, Bray-Curtis dissimilarities, and Haversine distances (with the geosphere package (59)) (see supplemental methods for details). Differential abundance was performed with ANCOM-BC (60)) with unrarefied phyloseq objects of hyporheic and SW samples separately, and with raw and continuous dTRC concentrations. The top 10 most significant (lowest *p*-values; all <0.05) ASVs in each log-fold change direction, associated with each dTRC, were used for further analysis. ANCOM-BC was also utilized to find ASVs that were differentially abundant with each sample type. Correlations analyses were done with metadata transformed with Tukey’s ladder of powers, to maximize linearity between variables (61) and normalized to an ordinal scale with the equation: [(value − min (value))/(max(value) − min(value))] for Spearman correlations with the corrplot package (62)). Shannon diversity index values for the correlations analysis were calculated with the microbiome package (63)) and raw richness measurements were taken by summing all ASV counts of differentially abundant ASVs from rarefied (see above) ASV count tables. dTRC concentration values were binned into “low,” “normal,” and “high” based on the 25th, 50th, and 75th percentiles, respectively, of each dTRC to assess how the relative abundance of ASVs changed by dTRC status. ASV relative abundance values by metadata grouping variables were visualized and identified with the Phinch2 desktop application (v2.0.1; (64)).

## RESULTS

### Physical characteristics of the river environment

Samples were collected from eleven stations on five rivers within the Sacramento River watershed at four time points designed to bracket fall-run Chinook salmon spawning (Fig. 1). Sample locations had a range of anthropogenic impacts. Heavily impacted sites were located on the Feather River, Clear Creek, and the Sacramento River. Sites on Battle Creek and Butte Creek were relatively less impacted; however, upstream water diversion structures and other channel modifications do exist on these rivers. One measure of the degree of anthropogenic control of each tributary was demonstrated by the observed river flow rates which showed natural rain-event-driven pulses in Battle, Butte, and Clear Creeks (Fig. 1). Major rain events occurred immediately before the Spawn (November) and Hatched (January) sampling events (Fig. 1). By contrast, elevated flow pulses were not seen in the Feather and Sacramento River sites where the flow is regulated by the upstream high head dams in a bimodal nature with high flow (670 and 7,000 CFS, respectively) before November and low flow (640 and 3,000 CFS, respectively) after November. The rain events between November and January caused increases in turbidity at the Spawn and Hatched time points that were especially evident downstream of Lake Oroville and Lake Shasta in the Feather River and Sacramento River, respectively, where turbidity reached 13.6 and 17.2 NTU, respectively (Fig. 1).

Temporal and regional patterns were observed in the water chemistry. Temperature across all stations dropped from a high median of 14.8°C in September to 8.9°C in January (Fig. 1). The Feather River was consistently the coldest and had the lowest variability in temperature of the tributaries with an observed temperature range of 9.2°C–10.4°C (Fig. 1). Conversely, the Battle and Butte Creek sites had the largest variability of observed temperatures with a range of 7.6°C–16.9°C. Chlorophyll-*a* concentration was significantly negatively correlated with temperature, with concentrations across all stations increasing from a median of 0.4 µg•L^−1^ in September to 0.71 µg•L^−1^ in January (Fig. 1 and 4). The Feather River sites had the highest observed chlorophyll-*a* concentrations ranging from 0.44 to 3.65 μg•L^−1^ (Fig. 1). Across all sites, median pH decreased from 7.88 in September to 7.5 in November, and then to 7.85 in January (Fig. 1). The Feather River had the lowest pH range across all time points (excluding outliers) running from 6.91 to 7.54, while all other tributaries had similar pH ranges across all time points ranging from 7.37 to 8.33 (Fig. 1). Dissolved oxygen concentrations increased from a median value of 9.44 mg•L^−1^ in September to 10.99 mg•L^−1^ in December before dropping to 10.65 mg•L^−1^ in January. No regional differences in dissolved oxygen were observed when samples were binned by tributary (Fig. 1).

### Distribution of dTRCs

dTRCs were detected in the femto-picomolar range in both the HZ and the SW of the Sacramento River watershed. Samples from all stations and time points were binned based on sample type (HZ and SW) to understand the basin-scale relationships between thiamine cycling in the HZ and SW (Fig. 2; Table 1). The B_1_ concentrations in both the HZ and SW were not significantly different (significant differences between HZ and SW means examined with paired *t*-tests; median values are 0.21 and 0.23 pM, respectively), and the interquartile ranges (IQR) of concentrations in the HZ and SW reflected this similarity with overlapping values of 0.14–0.29 pM and 0.17–0.35 pM (Fig. 2; Table 1). The concentrations of thiamine’s biosynthetic precursors, HMP and cHET, did not vary significantly but were higher in the HZ than in the SW (Fig. 2). The IQR of HMP in the HZ was 3.78–8.84 pM, whereas the SW IQR was 1.21–2.77 pM, and median HMP concentration was three times higher in the HZ than the SW. Similarly, the median concentration of cHET was 2.8 times higher in the HZ than in the SW. The IQRs for cHET in the HZ and SW were 133.67–546.33 pM and 38.22–219 pM, respectively (Fig. 2; Table 1). Opposingly to the findings of HMP and cHET, the median concentration of AmMP, a thiamine degradation product, was 3.5 times higher in the SW than in the HZ and the differences in means were significant (*p*-value = 0.0003; 8.25 and 2.33 pM, respectively). The AmMP IQR was 4.17–12.38 pM in the SW and 0.73–5.70 in the HZ. The IQRs of HET, both a thiamine degradation product and biosynthesis precursor, overlapped in the HZ and SW with values of 2.89–9.45 pM and 1.74–7.78 pM, respectively. The median of HET in the HZ was 4.64 pM and 3.14 pM in the SW. HET was frequently undetectable in the SW and was only detected in the Feather River and the South Fork of Battle Creek (Fig. S1; Table S1).

**TABLE 1:** Summary of HZ and SW dTRC picomolar concentrations^*a*^

	B_1_		HMP		AmMP		cHET		HET	
	HZ	SW	HZ	SW	HZ	SW	HZ	SW	HZ	SW
**Minimum**	0.10	0.06	1.24	0.45	0.16	0.61	54.73	16.67	1.33	1.02
**Lower quartile**	0.14	0.17	3.78	1.21	0.73	4.17	133.67	38.22	2.89	1.74
**Median**	0.21	0.23	5.20	1.77	2.33	8.25	222.50	79.53	4.64	3.14
**Upper quartile**	0.29	0.35	8.84	2.77	5.70	12.38	546.33	219.67	9.45	7.78
**Maximum**	0.76	22.30	227.67	418.33	18.63	1333.33	2623.33	3436.67	209.67	338.33

^*a*^ Data from this table is graphically presented in Fig. 2. The complete dTRC dataset is presented in Table S1.

Multiple statistical outliers (Tukey Test) were identified at both the high and low concentration extremes in the HZ and SW pools for all dTRCs (Fig. 2; Table 1). These outliers likely represent biologically significant events as has been previously established (26, 65). Further evidence for the biological relevance of these statistical outliers is that when concentrations are assessed on an individual station basis, South Fork Battle Creek was the source of the high extreme outliers in both the HZ and the SW at two separate sampling events: Spawn and Incubation (Fig. S1; Table S1).

**Fig 3:**
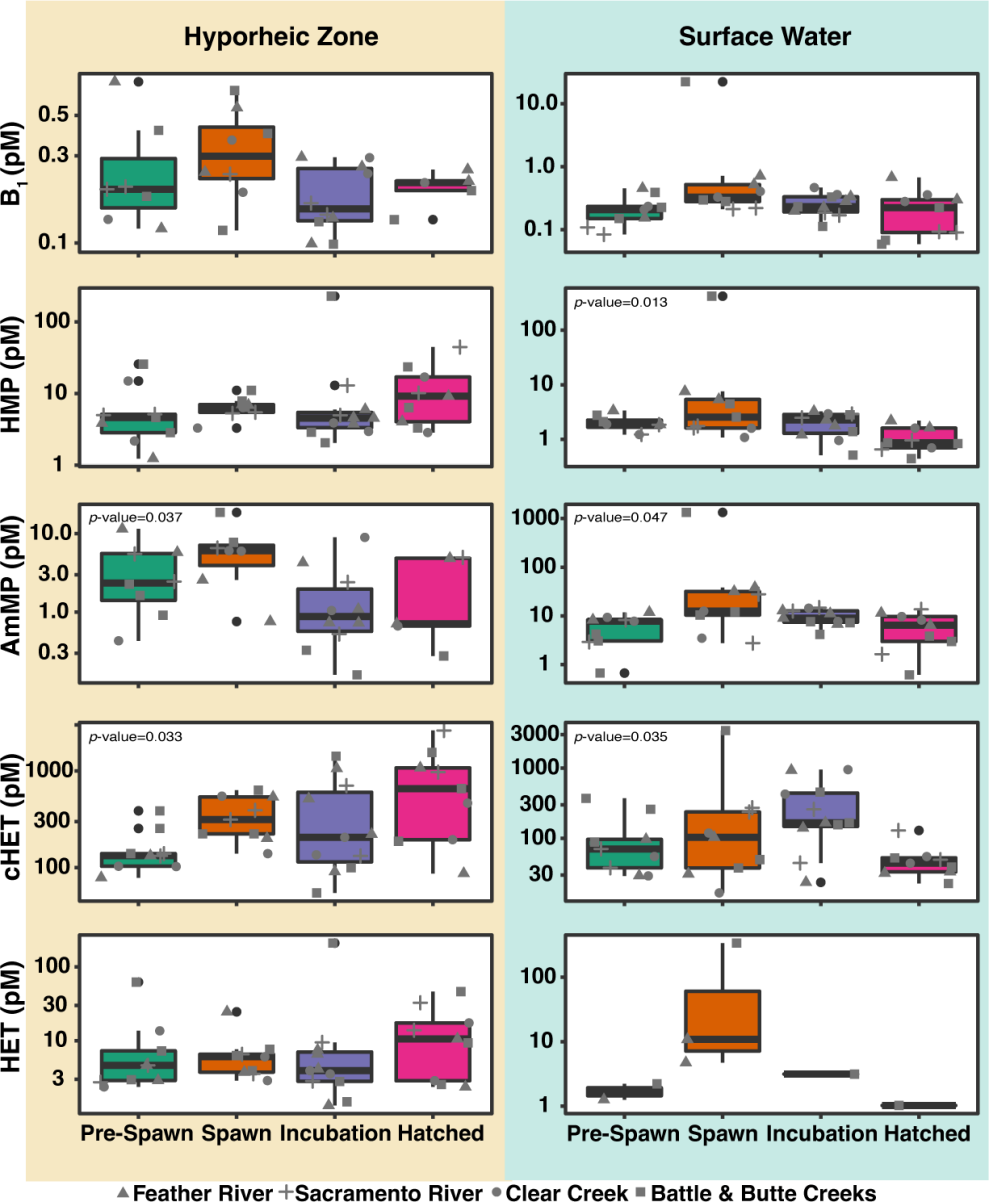
Temporal variations in dTRC concentrations. Box plots of Hyporheic Zone (tan) and Surface Water (blue) dTRC concentrations are binned by time point. Box colors represent sampling times. Individual points are overplotted and shapes indicate tributary: Feather River (triangle), Sacramento River (plus), Clear Creek (circle), and Battle and Butte Creeks (square). Kruskal-Wallis tests were used to test for significant differences in dTRC concentrations between time point bins; statistical significance is indicated where present.

We observed a substantial temporal pattern in dTRC concentrations in both the HZ and SW across all samples where the concentrations at Spawn were higher than the preceding and following sample points for all dTRCs but cHET (Fig. 3). Temporal variability in dTRC concentrations was assessed by binning samples from all stations by sample time point (Fig. 3). We determined that time point bins, rather than regional bins, were able to resolve a greater amount of the observed variation in dTRC concentrations as Kruskal-Wallis tests only show significant regional variability between regions for B_1_ in the SW (*p*-value = 0.0035), whereas this test found significant temporal variability for HMP in the SW (*p*-value = 0.013), AmMP in the SW and HZ (*p*-values = 0.047 and 0.037, respectively), and cHET in the SW and HZ (*p*-values = 0.035 and 0.033, respectively). In addition, we were able to qualitatively validate these results by comparing boxplot medians and IQRs which showed no clear regional trends but did show a temporal trend (Fig. 3; Fig. S1).

The temporal B_1_ trend we observed in the HZ showed an increase in median dTRC concentration from Pre-Spawn (0.2 pM B_1_, 4.63 pM HMP, 2.36 pM AmMP, 133.0 pM cHET, and 4.63 pM HET) to Spawn (0.31 pM B_1_, 6.43 pM HMP, 6.06 pM AmMP, 314.0 pM cHET, and 6.06 pM HET) followed by a decrease of median dTRC concentration at the Incubation time point (0.15 pM B_1_, 4.54 pM HMP, 0.89 pM AmMP, 206.0 pM cHET, and 3.93pM HET) (Fig. 3). The median concentration of all dTRCs except AmMP in the HZ at the Hatched time point increased from the Incubation time point (0.21 pM B_1_, 9.18 pM HMP, 0.71 pM AmMP, 657.67 pM cHET, and 10.57 pM HET). Despite AmMP having a lower median at the Hatched time point, the trend of increased concentrations holds as the AmMP IQR at Hatched (0.67–4.86 pM) was greater than the Incubation time point (0.58–2.07 pM).

Within the SW, a similar temporal trend in dTRC concentrations was observed in the HZ where the dTRC concentrations at Spawn were frequently the highest measured (Fig. 3). The median concentrations of all dTRC increased from Pre-Spawn (0.21 pM B_1_, 1.89 pM HMP, 7.76 pM AmMP, 70.57 pM cHET, and 1.47 pM HET) to Spawn (0.33 pM B_1_, 2.56 pM HMP, 12.50 pM AmMP, 103.27 pM cHET, and 10.84 pM HET). The median concentrations of all dTRCs except cHET decreased from Spawn to Incubation (0.23 pM B_1_, 2.26 pM HMP, 83.98 pM AmMP, 166.67 pM cHET, and 3.14 pM HET). All dTRCs saw a decrease in median concentration from the Incubation to Hatched time points (0.22 pM B_1_, 0.87 pM HMP, 6.45 pM AmMP, 44.13 pM cHET, and 1.02 pM HET).

### Relationship between dTRCs, microbial community diversity measures, and abiotic parameters

**Fig 4:**
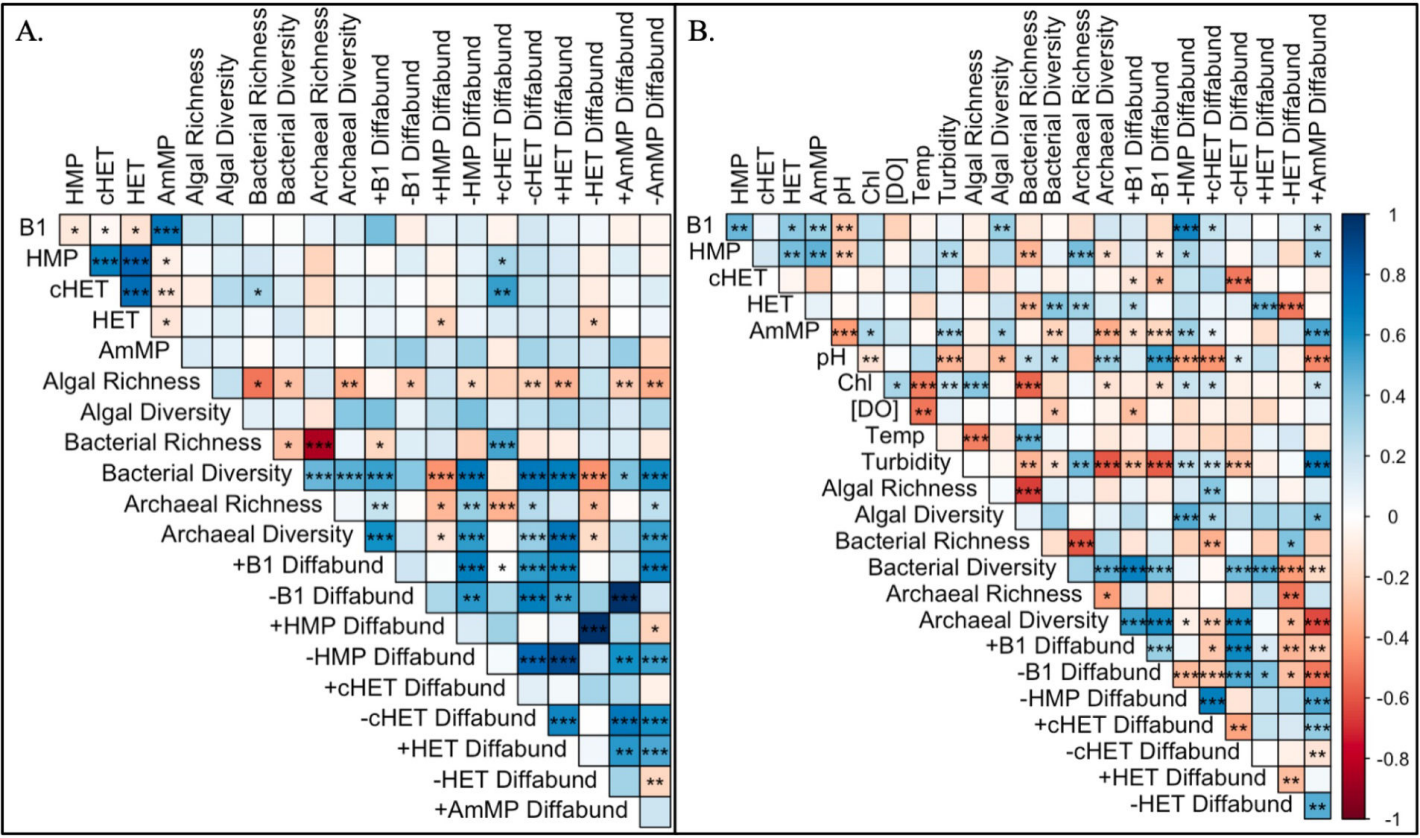
Correlogram of transformed and normalized A. HZ and B. SW dTRC concentrations, bacterial, archaeal, and algal (eukaryotic algae and Cyanobacteria) ASV richness and Shannon diversity (Diversity), and differentially abundant ASV richness with each dTRC. “+” and “−” dTRC “Diffabund” indicate ASVs that display positive and negative LFCs with dTRC concentrations, respectively. Positive correlations are depicted in shades of blue, while negative correlations are depicted in shades of red. Stars indicate statistically significant correlative relationships (*p* < 0.05).

Spearman correlations between dTRC concentrations (all correlations significant unless indicated otherwise) differed by sample type, indicating the potential for unique thiamine cycling in the SW and HZ. B_1_ concentrations in the HZ negatively correlated with HMP, cHET, and HET concentrations and showed positive correlations with AmMP (Fig. 4A). In the HZ, the biosynthetic precursors HMP and cHET positively correlated with each other, yet the degradation products, HET and AmMP negatively correlated (Fig. 4A). SW B_1_ concentrations correlated positively with HMP, HET, and AmMP (Fig. 4B). No significant negative co-correlations existed between dTRCs in the SW (Fig. 4B). Microbial Shannon diversity and abiotic parameters also uniquely correlated with dTRC concentrations by sample type. SW pH negatively correlated with concentrations of B_1_, HMP, and AmMP (Fig. 4B). Other SW abiotic factors including temperature, chlorophyll-*a*, and dissolved oxygen concentrations showed no significant correlations with dTRC concentrations, and turbidity positively correlated with HMP and AmMP concentrations (Fig. 4B). HZ bacterial richness positively correlated with cHET concentrations, and no other HZ microbial diversity matrices (richness and Shannon diversity) displayed significant associations with dTRC concentrations (Fig. 4A). In SW samples, algal Shannon diversity correlated positively with B_1_ and AmMP concentrations; yet, algal richness and chlorophyll-*a* did not display this trend (Fig. 4B). Bacterial SW richness negatively correlated with HMP and HET concentrations and bacterial SW Shannon diversity positively correlated with HET and negatively correlated with AmMP. Archaeal SW richness positively correlated with HMP and HET concentrations and archaeal SW Shannon diversity negatively correlated with HMP and AmMP concentrations (Fig. 4B). Notably, chlorophyll-*a* positively correlated with algal richness but not algal Shannon diversity.

**Fig 5:**
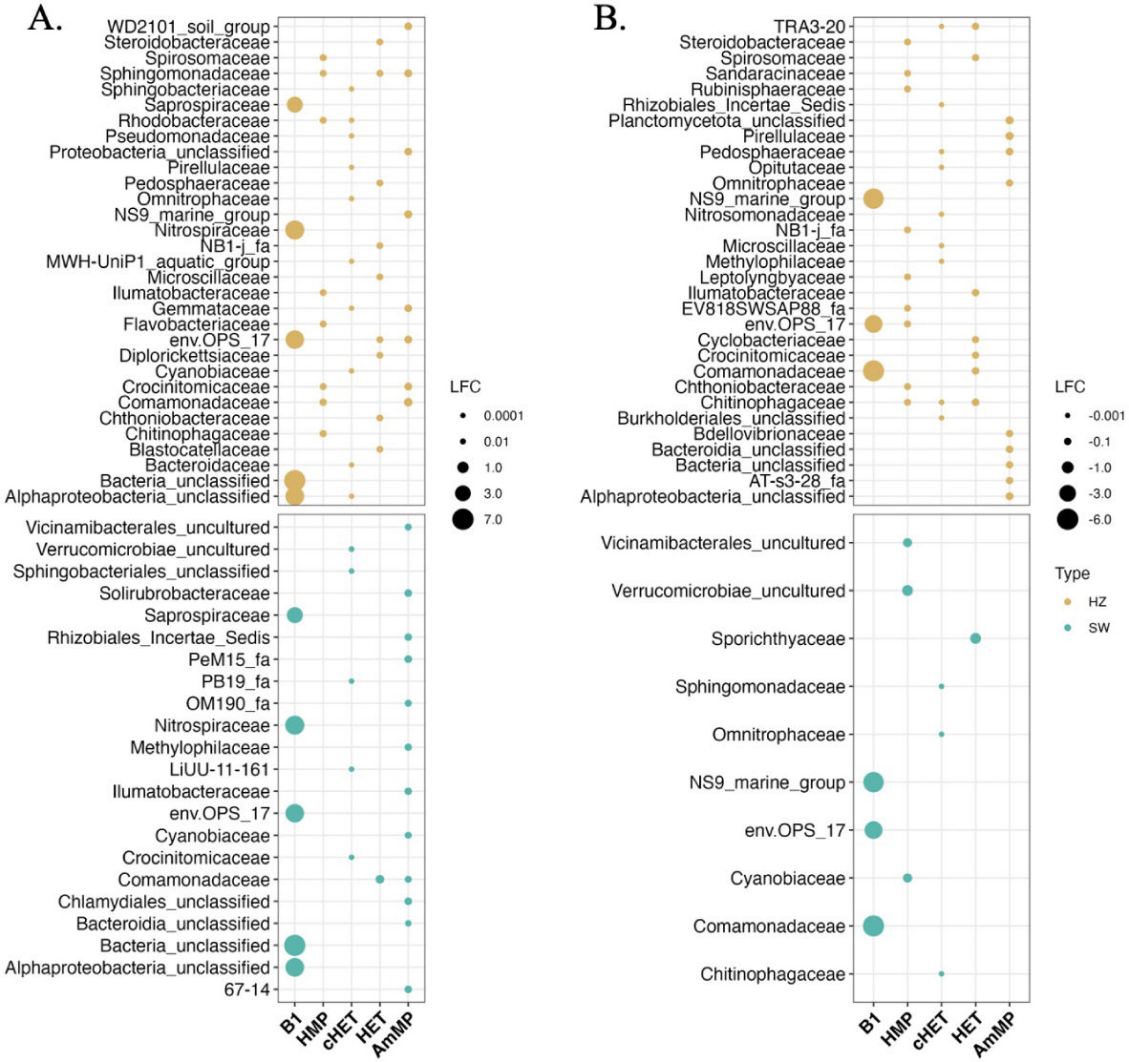
Bubble plot of (A) positive and (B) negative LFCs of differentially abundant ASVs. ASVs are significantly associated (*p* < 0.05) with raw dTRC concentration values. Each y-axis family represents the top 10 most differentially abundant ASVs per dTRC. Bubble color corresponds to sample type and the sizes of bubbles are related to LFC magnitudes.

Microbial richness, Shannon diversity, and the richness of differentially abundant ASVs correlated with dTRC concentrations to a higher degree in the WC than the HZ (Fig. 4). The direction of the Log-Fold Change (LFC; + or − direction) from differential abundance results displayed in Fig. 5 generally did not inform the direction of Spearman correlations (+ or −) between the richness of differentially abundant ASVs and dTRC concentrations, though some exceptions to this trend were present. In both sample types, the richness of +B_1_ differentially abundant ASVs correlated positively with B_1_ concentrations, and the opposite was true for −B_1_ differentially abundant ASVs, yet none of these correlations were significant (Fig. 4). The richness of SW −HMP, +cHET, and +AmMP differentially abundant ASVs all positively and significantly correlated with B_1_ concentrations (Fig. 4B). Significant Spearman correlations where the LFC direction of differentially abundant ASVs (Fig. 5) matched the Spearman correlation direction between differentially abundant ASV richness and dTRC concentrations included SW −cHET, +HET, −HET, and +AmMP (Fig. 4B) and HZ +cHET and −HET (Fig. 4A).

### Sacramento River microbial communities differ by diversity and sample site characteristics

**Fig 6:**
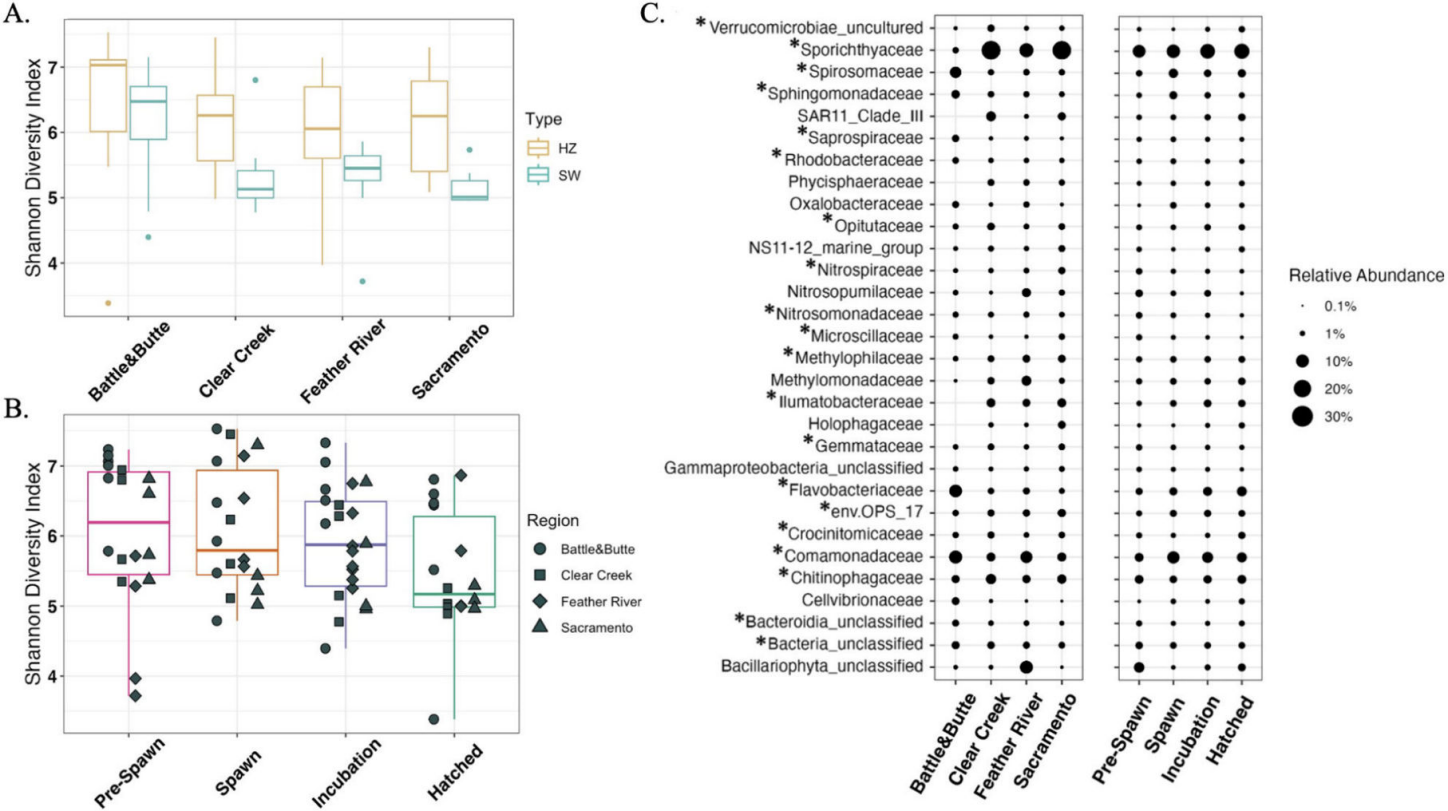
Microbial community diversity. Shannon diversity index values of bacterial, archaeal, and eukaryotic algal communities of SW and HZ samples binned by (A) region and (B) time point, and (C) relative abundance of ASVs displaying the top 30 most abundant families by region and time point. Boxes in (A) are colored by sample type and in (B) by time point. Bubble size in (C) represents relative abundance of specific ASVs and asterisks next to families represent those that contain species found to be differentially abundant with at least one dTRC.

HZ bacterial, archaeal, and algal communities displayed higher diversity (all diversity measured with the Shannon diversity index) than those of the SW in all Sacramento River watershed regions (Fig. 6). SW Battle and Butte Creek samples displayed the highest microbial diversity, followed by the Feather River, Clear Creek, and Sacramento River (Fig. 6A). HZ diversity was similarly higher in Battle and Butte Creeks than all other samples and the Feather River showed the lowest HZ diversity. Clear Creek and Sacramento samples displayed nearly identical medians (Clear Creek = 6.26; Sacramento = 6.25) in HZ diversity. Temporal trends in diversity were less apparent but displayed a slight decrease in medians across time points (Pre-Spawn = 6.20; Spawn = 5.80; Incubation = 5.88; Hatched = 5.17; Fig. 6B). Results from Kruskal-Wallis tests indicated HZ sample diversity significantly differed by time point (*p*-value = 0.014); yet, this was not true for SW samples (*p*-value > 0.05). SW samples significantly differed by region (*p*-value = 0.009) and HZ sample diversity did not significantly differ by region (*p*-value > 0.05).

Total rarefied bacterial and archaeal communities significantly differed by region, time point, and sample type (PERMANOVA *p*-values = 0.001, 0.018, and 0.003, respectively). CAP1 explained 22.1% of the variance and CAP2 explained 7.8% of the variance (Fig. S2). Significant differences in the variance of communities representing sample sites of each region also existed (permutest *p*-value = 0.0001), but not when samples were grouped by time point or sample type. Within-region variance was also highest in Feather River samples, based on the centroid size of these sites on the CAP ordination (Fig. S2). The bacterial and archaeal communities of Battle and Butte Creek differed greatly in composition from all other samples, especially on CAP1, and displayed low within-region variance (Fig. S2).

### Bacterial, archaeal, and algal community compositions are unique by sample type

The total rarefied community of both sample types included 17,733 bacterial, archaeal, and eukaryotic algal ASVs. A small number of abundant phyla dominated in relative abundances across both sample types and represented taxa from all three domains. These included Proteobacteria (SW: 31.7%; HZ: 33.7%), Bacteroidota (SW: 22.5%; HZ: 19.9%), Actinobacteriota (SW: 20.2%; HZ: 13.8%), Planctomycetota (SW: 4.3%; HZ: 6.1%), Verrucomicrobiota (SW: 4.6%; HZ: 5.7%), Acidobacteriota (SW: 2.3%; HZ: 3.6%), Ochrophyta (SW: 2.6%; HZ: 3.2%), Unclassified Bacteria (SW: 1.8%; HZ: 2.4%), Crenarchaeota (SW: 1.8%; HZ: 1.4%), and Bdellovibrionota (SW: 1.4%; HZ: 1.5%). Notably, a majority of the top most abundant families across sample sites were differentially abundant with at least one dTRC (20/30; Fig. 6C).

**Fig 7:**
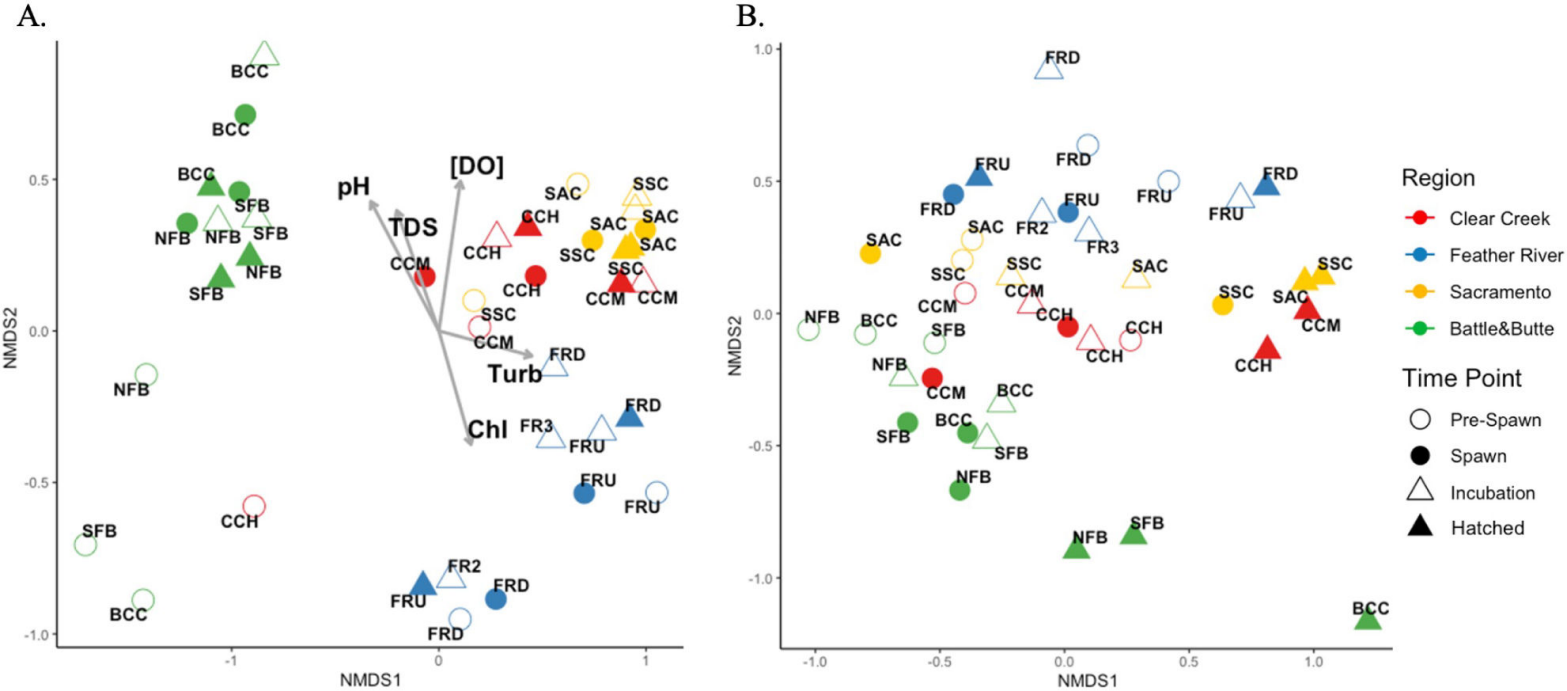
Bray-Curtis dissimilarity NMDS plots of (A) SW (stress = 0.1082) and (B) HZ (stress = 0.1219) bacterial, archaeal, and eukaryotic algal communities. Vectors display significant (*p*-value < 0.05) correlations between abiotic factors measured in the SW and sample site placements on the ordination. Points on the ordination are labeled by station, colored by region, and shapes correspond to time points. [DO] = dissolved oxygen (mg/L); Chl = Chlorophyll *a*; TDS = total dissolved solids; Turb = turbidity.

Among the abiotic factors that were measured, vectors (each vector *p*-value < 0.05) associated with water turbidity and chlorophyll-*a* concentration aligned with the distribution of Feather River SW samples in NMDS space. The concentration of dissolved oxygen significantly influenced the placement of SW Clear Creek and Sacramento sites and total dissolved solids and pH influenced both Battle and Butte Creek and Sacramento River samples (Fig. 7A). dTRC concentrations did not significantly influence (each vector *p*-value > 0.05) the placement of any samples of either ordination (lack of dTRC vectors on Fig. 7). Significant differences existed between SW communities by region (PERMANOVA *p*-value = 0.001) but not by time point (*p*-value > 0.05). Significant differences in within-group variance existed when SW samples were grouped by region (permutest *p*-value = 0.001) but not when SW samples were grouped by time point (*p*-value > 0.05).

Significant differences existed between HZ samples by both region (PERMANOVA *p*-value = 0.001) and time point (PERMANOVA *p*-value = 0.011), and no significant differences existed between within-group variance of either variable in the HZ (permutest *p*-value > 0.05). Battle and Butte Creek samples displayed similarities in community compositions regardless of both sample type and time point (Fig. 7). No discernable patterns were observed for SW sample communities when grouped by time point (Fig. 7A). In particular, the Sacramento River stations, SSC and SAC clustered closely together independent of time point (Fig. 7A). All Battle and Butte Creek HZ microbial communities displayed high similarity by each of the time points and Clear Creek and Sacramento samples displayed similarities during the Hatched time point (Fig. 7B). Mantel test results indicated both WC and HZ sample community dissimilarity significantly increased as a function of increased geographic (Haversine) distance between sample sites (SW: Mantel test statistic = 0.272; *p*-value = 0.0002; HZ: Mantel test statistic = 0.241; *p*-value = 0.0002). Community dissimilarity was not significantly associated with any dTRC concentrations (*p*-value > 0.05) in the HZ. SW community dissimilarity was significantly associated with SW HMP concentrations (Mantel test statistic = 0.233; *p*-value = 0.017) and, to a lesser degree, with HET concentrations though this was not significant (Mantel test statistic = 0.173; *p*-value = 0.065).

### ASV relative and differential abundances are linked to dTRC concentrations

ANCOM-BC results indicated associations (all significant: *p*-value < 0.05) between ASVs in SW and HZ samples and every dTRC, especially B_1_ (+ and – LFCs; Fig. 5). Families of differentially abundant ASVs in positive and negative LFC correlations with B_1_ exhibited similar relative abundances between SW and HZ samples, and included Saprospiraceae (SW: 1.06%; HZ: 0.77%), Nitrospiraceae (SW: 0.78%; HZ: 0.91%), ENV OPS-17 (Sphingobacteriales Order; SW: 2.18%; HZ: 2.14%), Unclassified Bacteria (SW: 1.77%; HZ: 1.82%), Unclassified Alphaproteobacteria (SW: 0.41%; HZ: 0.52%), NS9 marine group (Flavobacteriales order; SW: 0.08%; HZ: 0.12%), and Comamonadaceae (SW: 6.88%; HZ: 5.71%) (Fig. 6C).

Thiamine biosynthetic precursor compounds (cHET and HMP) and degradation products (HET and AmMP) showed much lower LFC magnitudes than B_1_ (Fig. 5). Families of ASVs that were both differentially abundant in the HZ and with dTRC concentrations included Nitrospiraceae, Blastocatellaceae, Methylophilaceae, Uncultured Bacteria, Pedosphaeraceae, Rhizobiales *incertae sedis*, Chitinophagaceae, and Comamonadaceae (Fig. S3; Fig. 5). Only two families of ASVs, Saprospiraceae and Leptolyngbyaceae, were both differentially abundant in the SW and with dTRC concentrations (Fig. S3; Fig. 5). A sum of 342 ASVs were differentially abundant in association with each dTRC in HZ samples and a sum of 44 ASVs were differentially abundant in association with each dTRC in SW samples (grouped into each Fig. 5y-axis family).

The relative abundance of ANCOM-BC-identified ASVs displayed unique trends in samples grouped by region and binned dTRC concentrations (see methods). Samples grouped into the low B_1_ bin contained high relative abundances of Flavobacteraceae (4.45%) compared to Flavobacteraceae in normal (3.69%) and high B_1_ bins (3.07%), although all three bins still displayed high relative abundances of Flavobacteraceae. All other differentially abundant taxa displayed numerically even relative abundances across B_1_ concentration bins. Sporichthyaceae and Ilumatobacteraceae ASVs spiked in relative abundance in samples grouped into the low HET bin (17.56% and 3.19%, respectively) and were much lower in relative abundance in the normal (8.90% and 1.47%, respectively) and high (7.99% and 1.33%, respectively) HET bins. Similar to HET, the low HMP bin spiked in the relative abundance of Sporichthyaceae (16.19%) ASVs, whose relative abundance decreased in normal (12.38%) and high HMP (7.88%) bins. Opposingly, Sporichthyaceae ASVs spiked in relative abundance in samples grouped into the high AmMP bin (16.75%) and decreased in relative abundance in the normal (11.71%) and low (8.65%) AmMP bin.

Clear regional trends in the relative abundances of families of ANCOM-BC-identified ASVs were apparent. Butte and Battle Creek samples contained high relative abundances of Flavobacteriaceae, Spirosomaceae, and Comamonadaceae ASVs (8.58%, 6.47%, and 9.06%, respectively; Fig. 6C) compared to the Feather River (1.86%, 1.49%, and 7.39%, respectively; Fig. 6C), Clear Creek (1.43%, 1.26%, and 3.49%, respectively; Fig. 6C), and Sacramento (1.11%, 1.35%, and 3.59%, respectively; Fig. 6C). The opposite was true for Ilumatobacteraceae and Sporichthyaceae, which displayed high relative abundances in the Feather River (2.59% and 10.84%, respectively; Fig. 6C), Clear Creek (3.37% and 21.29%, respectively; Fig. 6C), and Sacramento River (3.44% and 20.93%, respectively; Fig. 6C) compared to Battle and Butte Creek samples (0.05% and 1.36%, respectively; Fig. 6C). Although diatoms were not identified as being differentially abundant with dTRC concentrations, it is notable that Feather River sample sites contained high relative abundances of Unclassified Bacillariophyta ASVs (diatoms; 8.39%) and these ASVs were found in relative abundances below 1% in all other regions (Fig. 6C). SAR11 clade III and CL500-3 (Phycisphaeraceae) were also higher in relative abundances in Sacramento River, Feather River, and Clear Creek samples than Battle and Butte Creek samples (Fig. 6C) and in low HET and HMP bins than normal and high bins. SAR11 clade III taxa had relative abundances of 2.54%, 1.11%, and 1.00% in low, normal, and high HET bins, respectively, and CL500-3 taxa had relative abundances of 1.23%, 0.88%, and 0.69% in low, normal, and high HMP bins, respectively.

## DISCUSSION

The increase in TDC in freshwater systems, and the Sacramento River watershed specifically, has led us to investigate the spatial and temporal distribution of dTRCs and the related microbial communities in the SW and HZ of the Sacramento River and its tributaries (Fig. 1) (1, 66). We determined the spatial and temporal distributions of dTRCs and the related microbial communities in the Sacramento River watershed in the SW and HZ (Fig. 1). Environmental thiamine chemistry is a new and rapidly developing field where much remains to be discovered. Simultaneous, direct dTRC measurements have never been conducted in freshwater systems and yet such an approach is the best way to obtain a synoptic understanding of the availability of this key vitamin. Thiamine has been infrequently measured previously in aquatic and estuarine systems (33–35). Distributions of dTRCs in marine systems have been studied more extensively, yet only roughly 500 discrete measurements have been published; thus, we are still assembling fundamental ecological knowledge regarding these metabolites required by all life. A recent report from the North Atlantic observed dTRC concentrations to be within the picomolar range (26). Rate-based measurements of thiamine cycling (e.g., biological uptake, excretion, abiotic degradation) are even more sparse due to the complexity of the assays, which make the interpretation of the available standing-stock dTRC concentrations challenging (67). Measured concentrations of dTRCs in environments are based on the equilibrium between microbial biosynthesis, dTRC excretion from cells due to lysis by viruses or grazers, microbial uptake of TRCs from the dissolved pool, and dTRC degradation by temperature, pH, UV light, and enzymatic activity (68). In this study, we used an inference-based approach, pairing microbial community composition analysis with dTRC measurements to investigate the role of river and HZ microbial communities in the cycling of dTRCs, with the ultimate goal of making linkages to thiamine deficiency complex in Chinook salmon.

### Thiamine cycling differs in marine and freshwater systems

The differences between the observed dTRC concentrations in the Sacramento River and those previously reported in marine systems suggest that the thiamine cycle in each environment is controlled by a unique set of processes. B_1_ concentrations observed in the Sacramento River SW and HZ (Fig. 2; Table 1) are between two and four orders of magnitude lower than what has been reported in marine environments. Within the marine water column, the interquartile range of dissolved concentrations of B_1_ ranges between 15 and 64 pM (only ca. 500 discrete marine dissolved B_1_ measurements exist) (26). The interquartile range of marine porewater B_1_ ranges between 237 and 462 pM; B_1_ concentrations have only been measured in one study where deep ocean sediment pore water (890 m water depth) was collected over the complete redox zonation ranging from oxic to anoxic (40). The HZ samples in this study are from unconsolidated, oxic, river gravels; thus, the comparison between these sample types is imperfect. The interquartile concentration ranges of both the pyrimidine compounds HMP and AmMP from this study (Fig. 2; Table 1) overlap those previously reported in the marine environment which are 0.3–5.1 pM HMP and 5.3–12.1 pM AmMP (26, 39, 45, 65). The thiazole moieties cHET and HET are both reported to be present at lower concentrations in the marine environment than in the Sacramento River SW where median concentration values were 8.4 and 11.3 times higher, respectively (26) (Table 1). The authors are not aware of any published marine or freshwater sediment-associated HMP, AmMP, cHET, or HET concentrations.

It is unclear what factors drive the substantial observed differences between marine and Sacramento River dTRC concentrations. Physical and chemical differences between oceans and rivers could contribute to the observed concentration disparities as thiamine is known to abiotically degrade at high pH levels and with UV light (69–71), which could impact river SWs more readily than the marine water column. In addition, the differences in mixing and advective processes between rivers and the ocean could have a major impact on dTRC concentrations. Furthermore, the role of the distinct microbial communities in marine and freshwater environments cannot be discounted as biological activity (e.g., dTRC uptake and production) contributes to the standing stocks of each of these compounds in each environment. The disparities that we observed between marine and freshwater dTRC concentrations suggest that findings from investigations on thiamine cycling in marine ecosystems could be poor predictors of freshwater thiamine dynamics. Future research will be required to fully understand the factors controlling thiamine cycling in marine and freshwater systems.

### dTRC distributions in the Sacramento River system

All dTRCs were detected in both the SW and HZ of the Sacramento River system (Fig. 2; Fig. S1; Table 1; Table S1). This is the first report on the distribution of all these compounds in aquatic environments. The concentrations of B_1_ across the entire watershed were exceedingly low, with median concentrations in the sub-picomolar range (0.21 pM HZ, 0.23 pM SW). B_1_ concentrations were the lowest of the dTRCs measured in this study (Fig. 2; Table 1). We hypothesize that B_1_’s low concentration is due to a combination of its abiotic instability and its biological importance, which would drive rapid B_1_ uptake (microbial and metazoan) from the extracellular environment. Microbial community results support this hypothesis because differentially abundant ASVs had more measurable impacts on B_1_ than all other dTRCs regardless of sample type (LFC magnitudes in Fig. 5). The known cellular enzymatic half-saturation constants for thiamine are in the nanomolar range and indicate that cells must have high-affinity uptake mechanisms for this coenzyme (72). Thiamine auxotrophy (i.e., obligate requirement) is known to be prevalent in many groups of abundant and environmentally important microorganisms which creates a robust demand for dissolved thiamine and its congeners in most environments (22, 38, 39). In addition, we observed that the B_1_ degradation product AmMP was present at concentrations between one and two orders of magnitude higher than B_1_. These data taken together suggest that microbial activity coupled with abiotic degradation could be responsible for driving equilibrium B_1_ concentrations (e.g., the balance between production, uptake, and degradation) into the femtomolar range.

The measured concentrations of HMP and cHET which are the biosynthetic precursors for B_1_, are between two and three orders of magnitude greater than B_1_ (Fig. 2; Table 1). These data do not support the alternate hypothesis that low B_1_ concentrations are due to low rates of production and instead provide substantial evidence for relatively high rates of thiamine biosynthesis in both the SW and the HZ. The sole known source for HMP and cHET is the thiamine biosynthesis pathway (25); therefore, it is somewhat surprising to see these intracellular tracers of the thiamine biosynthesis pathway in the extracellular dissolved pool. However, multiple studies have reported these compounds to be present in spent culture media and the environment, and have implicated cellular leakage, excretion, cellular lysis, and sloppy feeding have been implicated as mechanisms for their release (26, 38, 39, 68). Future investigations will be required to quantify the rates at which each dTRC is produced and removed to fully constrain the freshwater thiamine cycle.

We observed that biosynthetic precursors of thiamine, HMP and cHET, are in higher concentrations in the HZ than in the overlying SW which we interpret to be evidence for active thiamine biosynthesis in the HZ (Fig. 2). This observation provides the strongest evidence to date that the HZ is source of dTRCs to the SW, building on previous research from marine and freshwater systems (35, 40). Riverine HZs are a major source of microbes and metabolites to the overlying river, and the residence time of the water in the river is directly proportional to the abundance and diversity of both microbes and metabolites (73, 74). It is not clear how variations in river flow influence dTRC flux from the sediments but changes in HZ residence time likely influence this process. Additional abiotic and biotic factors such as organic carbon loading, sediment particle size, and discharge rate, which impact permeability and the extent of the HZ and its exchange with the SW (75), likely influence dTRC SW concentrations.

Previous research has shown that thiamine bath treatments of Atlantic salmon eggs and sac-fry can prevent TDC-driven mortality (76). Although the thiamine concentrations in these treatments are substantially higher than our measured values, our data show that a natural dissolved thiamine pool does exist and could supplement the early life stages of Chinook salmon. Our findings are therefore of special interest, as a naturally occurring source of thiamine in spawning gravels could mitigate symptoms of TDC as eggs and sac-fry absorb thiamine over time.

### Regional variability in dTRC concentrations

Despite observed regional variations in river physical and chemical parameters, only weak regional differentiation in dTRC concentrations was observed (Fig. 1 and 2; Fig. S1). Sampling locations for this study were chosen to assess the impact of a gradient of anthropogenic impacts and salmon spawning success on dTRC availability. We initially hypothesized that due to these varying impacts, dTRC concentrations would vary based on river region. This hypothesis was supported by our physical and chemical data from these rivers which demonstrate differing hydrological regimes in tributaries below high-head dams and those with lower levels of anthropogenic impacts (Fig. 1). For example, multiple precipitation events occurred during our sampling period which caused pulses of high flow in Battle, Butte, and Clear Creeks (Fig. 1); however, these flow events were prevented by upstream dams at our sites in the Sacramento and Feather Rivers. The existence of the Oroville dam upstream of the Feather River sampling sites may have contributed to the strong regional signature observed at these sites, where the pH and temperature were consistently lower than other tributaries while the turbidity and chlorophyll-*a* were consistently higher (Fig. 1). Spearman correlations showed that pH negatively correlated with SW B_1_, HMP, and AmMP concentrations, yet did not impact cHET and HET (Fig. 4). The consistently lower pH at the Feather River sites, resulting in increased chemical stability of dTRCs, likely caused the slight observed increase in B_1_, HMP, and AmMP concentrations in the Feather River SW relative to other regions (Fig. 1; Fig. S1). Kruskal-Wallis tests showed that B_1_ within the surface water differed significantly by region (*p-*value < 0.05). This increase in dTRC concentrations at the Feather River sites may have been limited by the higher abundances of potentially auxotrophic algae at this site as evidenced by higher chlorophyll-*a* concentrations and diatom ASV relative abundances (Fig. 1, 6C) (36). These observations demonstrate the complexity of thiamine cycling, where multiple variables impact both the production and removal processes of dTRCs.

### Temporal influences on dTRC concentrations

Temporal patterns of dTRC concentrations across the sample sites were observed which suggest that there could be linkages between microbial dTRC production and the Chinook salmon life cycle (Fig. 3). There was a clear seasonal signature observed in our chemical data where temperature across all sample sites decreased from September to January (Fig. 1). In addition, we observed that chlorophyll-*a* and dissolved oxygen increased over this same time period (Fig. 1). These trends reflect the seasonal progression from late summer to mid-winter. However, when the dTRC concentrations are binned by sample time point they show a temporal pattern for all dTRCs that is decoupled from seasonality (Fig. 3). We observed that maximum dTRC concentrations generally occurred at the Spawn (November) time point which occurred while the fall-run Chinook salmon were actively spawning in the Sacramento River watershed (Fig. 3). The Spawn maximum was generally followed by a decrease in dTRC concentration at the Incubation (December) time point which occurred while the fall-run Chinook salmon eggs were incubating in the river gravels (Fig. 3). At the final sample point Hatched (January), we assume that most fall-run Chinook salmon eggs that were in the gravel at the Incubation time point would have hatched and it is likely that the juveniles were still benthically associated (44). At the Hatched time point in the HZ, dTRC concentrations increased, while in the SW they decreased (Fig. 3). Kruskal-Wallis tests showed that these temporal differences were statistically significant for HMP in the SW and AmMP and cHET in both the SW and HZ (*p*-value < 0.05).

The processes driving the observed temporal trend in dTRC concentrations across our sample sites are not clear; however, our data do indicate that these processes are decoupled from the seasonal changes occurring in water chemistry (Fig. 1C). Research has indicated that spikes in dTRC concentrations in marine environments are frequently related to shifts in the microbial community composition and activity (26, 65). In this study, we observed that microbial communities in the HZ differ by sample time point, as opposed to the SW (Fig. 7), providing evidence for a stronger association between microbial community compositions and dTRC concentrations in the HZ than in the SW.

### Human impacts on rivers influence microbial community compositions

The compositions of Sacramento River watershed microbial communities showed small-scale homogeneity and large-scale heterogeneity. The diversity of microbial communities can be dependent on both river reach (77, 78) and sample type (79). High similarity between microbial communities of stations in the same river region was observed and indicated a lack of evidence for river reach driving community heterogeneity (Fig. S2). Regions with high anthropogenic impact (e.g., Sacramento, Feather River, and Clear Creek) also displayed more within-region variance than Butte and Battle Creek sites (Fig. S2), providing evidence that anthropogenic impacts could increase microbial community dissimilarity between locations along the same river and between the surface water and hyporheic zone. This could result in sporadic compositions of microbial communities along the spectrum of human-impacted rivers where Chinook salmon spawn. Human-constructed dams and the resulting reservoirs impede river flow and can alter microbial diversity in fluvial systems (74, 79, 80). Although our results do not contain above- and below-reservoir sample locations, we did observe differences between sites downstream of dam-formed reservoirs and sites with lower human impact. Battle and Butte Creek sites displayed higher microbial diversity in both sample types than heavily impacted river sites (Fig. 6A), which provides evidence that anthropogenic impacts limit microbial diversity in the Sacramento River watershed.

### Microbial diversity and community compositions influence dTRC concentrations uniquely by sample type

While SW Shannon diversity differed by river region, HZ Shannon diversity differed only by time point in a similar manner to dTRC concentrations. However, few significant correlations existed between HZ Shannon diversity and dTRC concentrations (Fig. 4A). Together, these results provide evidence that HZ microbial diversity changed in accordance with temporal variation but did not greatly influence dTRC concentrations. River HZs are environments of high chemical complexity and microbially driven biochemical and respiratory activity (81). High-level measurements of microbial diversity, therefore, may not reflect the biochemical complexity of this environmental system, whereas changes in the relative abundance of individual ASVs could influence small-scale biochemistry, and therefore dTRC concentrations.

Contrary to the HZ, microbial diversity and the availability of dTRCs in the SW appear to be related (Fig. 4B). Far more significant correlations were observed between SW microbial richness and Shannon diversity and dTRC concentrations, providing evidence that microbial diversity could be an indicator for dTRC availability in the SW. Microbial community compositions displayed regional signatures in both sample types (Haversine distance Mantel test results and Fig. S2; Fig. 7), yet only HZ samples displayed community compositions unique to salmon spawn time points. This finding provides evidence that the structure of microbial communities in the HZ, influenced by the relative abundances of certain ASVs, could impact dTRC concentrations in occurrence with the Chinook salmon life cycle.

### Relationship between microbial taxa and dTRCs

Microbially driven thiamine cycling is likely more complex in the HZ than water column based on the high numbers of differentially abundant ASVs with HZ dTRC concentrations, compared to those in the SW. The larger LFC magnitudes of differentially abundant ASVs associated with B_1_, compared to the other dTRCs, give evidence for intact thiamine availability being more highly influenced by the presence of certain taxa than the other dTRCs. The observation that a majority of taxa with high relative abundances influence dTRC concentrations (Fig. 6C) also suggests that abundant microbes may influence SW and HZ dTRC availability to a high degree compared to rarer taxa.

SW algal Shannon diversity corresponded to SW dissolved B_1_ availability to a higher degree than the relative abundance of particular algal taxa, algal richness, and chlorophyll-*a* concentrations. Evidence for this finding was indicated by diatom ASVs being found in high relative abundances in Feather River samples; yet, these taxa were not differentially abundant with any dTRC concentrations (Fig. 4B). These results follow previous findings, which have implicated the existence of algal blooms (low algal diversity states) with depleted levels of dissolved thiamine (36, 82). Conversely, individual bacterial taxa appeared to greatly influence dTRC concentrations. ASVs of several different families were found to be differentially abundant in both sample types and in both LFC directions with multiple dTRCs, especially B_1_, and included ENV OPS-17, Comamonadaceae, Unclassified bacteria (unclassified to phylum level), and NS9 marine group (Fig. 5). ASVs within each of these families may therefore display site-specific thiamine-cycling characteristics influenced by unknown extracellular conditions.

Our data demonstrate that human-impacted freshwater habitats harbor heightened relative abundances of bacteria that negatively influence thiamine availability than habitats with less human impact, which could influence the availability of B_1_ to Chinook salmon. Sporichthyaceae ASVs captured the above trend and the hgcl clade (genus annotation of Sporichthyaceae) was the most abundant genus across all sample sites yet were exceedingly low in relative abundances in Battle and Butte Creeks (Fig. 6C). Hgcl clade taxa have previously been associated with eutrophic aquatic settings, which could correspond to diatom overabundances that could indicate algal bloom states (83). These ASVs also displayed heightened relative abundances in sample sites binned into low HMP and HET, yet no change in relative abundance in B_1_ bins, indicating evidence for precursor auxotrophy, drawdown, and limited thiamine biosynthesis. In addition to hgcl clade, several other genera exhibited higher relative abundances in high human-impact sites compared to Battle and Butte Creeks and included CL500-3 (Phycisphaeraceae; Fig. 6C), CL500-29 marine group (Ilumatobacteraceae; Fig. 6C), and SAR11 clade III (Fig. 6C). Each of these taxa displayed heightened relative abundances in low sample bins of HET and HMP. Furthermore, CL500-3, CL500-29, and hgcl genera have been found in high relative abundances in reservoirs of past studies (84, 85). This provides limited evidence that human-made reservoirs seeding fluvial systems with bacteria which could draw down concentrations of certain dTRCs (HET and HMP) and potentially lack the genes necessary for thiamine biosynthesis.

Gene activity needs to be assessed to link the transcription (or lack of transcription) of thiamine biosynthesis and salvage genes to dTRC environmental concentrations. Although we were able to correlate ASV compositions with dTRC concentrations, the activity of thiamine-related gene pathways ultimately dictates the availability of these compounds to the environment through microbial production and consumption of thiamine-related compounds. Previous research in marine settings also show that microbial communities existing in structural disequilibria display heightened auxotrophy, limiting overall thiamine availability (26). The anthropogenic impact is also known to limit the availability of thiamine to higher trophic organisms due to nutrient input and climate change factors (23). Linking human impact, which could increase microbial community disequilibria, with the transcription of thiamine-related genes and the concentrations of dTRCs in fluvial systems is therefore of future relevance to assess the drivers of thiamine availability in Chinook salmon spawning habitats with differing degrees of anthropogenic disturbances.

We observed that heavily human-impacted freshwater systems harbor microbial communities with diminished microbial diversity and unique community compositions, increased community dissimilarity between sites within the same river region, and higher relative abundances of taxa negatively associated with dTRC concentrations. River systems heavily impacted by human pollution have been previously shown to contain unique microbial community structures and diversity compared to pristine fluvial habitats (86). Several studies have also displayed the many negative health outcomes anthropogenic pressures, including pollutants, habitat loss, climate change, freshwater acidification, and the building of dams, have on salmon in marine and freshwater systems (87–90). Linking microbial community compositions and diversity to dTRC concentrations in rivers where salmon spawn that are heavily human impacted is of great importance to obtain a comprehensive trophic view of TDC in natural fluvial systems. Although more detailed research is required, our data suggest that the availability of thiamine to spawn Chinook salmon, incubating eggs, and hatched fry may therefore be constrained in fluvial settings impacted by human-made dams and other anthropogenic stressors.

### Linking dTRCs, microbial communities, and salmon: developing a hypothetical framework

Our understanding of freshwater thiamine dynamics and their relationship to salmon TDC is at an early stage of discovery. However, our observations from this study allow for the construction of a preliminary hypothetical framework that can be used to guide future TDC research. We determined that dTRCs are present in the picomolar range in both the HZ and SW of the Sacramento River system. The distributions of thiamine’s biosynthetic precursor moieties allow us to conclude that the HZ is likely a source of dTRCs to the overlying SW. This finding is of special interest as the HZ is the habitat where the early life stages of salmon, which may be suffering from TDC, incubate. Furthermore, we observed temporal variation in dTRC distributions such that the peak in dTRC concentration aligns with the presence of spawning salmon, which is followed by a decrease in concentration while the early life stages of salmon are incubating in the river gravels. While our study does not provide evidence to indicate that this observed decrease in dTRC concentration was caused by salmon-related uptake, previous research has shown that early life stages of salmon (eggs and embryos) can assimilate dissolved thiamine from the environment (21). Such a relationship between high dTRC concentrations and salmon spawning could serve to mitigate the impact of maternally acquired thiamine deficiency complex on eggs and embryos. The mechanisms driving our observations remain undefined; however, they could be linked to spawning-induced benthic remodeling (91–94) causing changes in the residence time of HZ water resulting in a release of thiamine. In this way, salmon would be functioning as ecosystem engineers by digging redds, creating an environment rich in dTRCs which could directly increase the rates of survival of their progeny. It remains to be demonstrated that there is a causal link between salmon presence and dTRC availability, or that this pattern is repeatable over interannual cycles. Regardless, these data are an intriguing first step into understanding the connection between thiamine dynamics and microbial communities of freshwater, and lotic systems, and how this association may impact spawning Chinook salmon.

## Data Availability

The molecular datasetsdata sets presented in this study can be found in the NCBI sequence read archive (95). PRJNA996220. All dTRC data are published in Table S1. All scripts used to process the data are posted at GitHub.

## SUPPLEMENTAL MATERIAL

Supplemental material aem.01760-23-s0001.pdf Supplemental methods, figures, and table.

ASM does not own the copyrights to Supplemental Material that may be linked to, or accessed through, an article. The authors have granted ASM a non-exclusive, world-wide license to publish the Supplemental Material files. Please contact the corresponding author directly for reuse.
