# Supplementary material for: Connecting thiamine availability to the microbial community composition in Chinook salmon spawning habitats of the Sacramento River basin
Source: Appl Environ Microbiol. 2023 Dec 12;90(1):e01760-23. doi: 10.1128/aem.01760-23 (PMC10807462; doi:10.1128/aem.01760-23)
Supplement: Supplemental material — Supplemental methods, figures, and table. [file aem.01760-23-s0001.pdf]

Connecting Thiamine Availability to the Microbial Community Composition in Chinook Salmon Spawning Habitats of the Sacramento River Basin

Running Title:
Sacramento River Thiamine

Suffridge, Christopher P.[*+], Oregon State University, Department of Microbiology. 226 Nash Hall, Corvallis, OR 97331
Shannon, Kelly C.[*], Oregon State University, Department of Microbiology. 226 Nash Hall, Corvallis, OR 97331
Matthews H., Oregon State University, Department of Microbiology. 226 Nash Hall, Corvallis, OR 97331
Johnson R., NOAA Fisheries, Southwest Fisheries Science Center, Fisheries Ecology Division, 110 McAllister Way, Santa Cruz, CA 95060 and University of California Davis, Center for Watershed Sciences, 1 Shields Ave. Davis, CA 95616
Jeffres C., University of California, Davis, Center for Watershed Sciences, One Shields Ave, Davis 95616
Mantua N., NOAA Fisheries, Southwest Fisheries Science Center, Fisheries Ecology Division, 110 McAllister Way, Santa Cruz, CA 95060
Ward, Abigail E., University of California, Davis, Center for Watershed Sciences, One Shields Ave, Davis 95616
Holmes E., University of California, Davis, Center for Watershed Sciences, One Shields Ave, Davis 95616 AND California Department of Water Resources, 3500 Industrial Blvd. West Sacramento, CA 95691
Kindopp, J., California Department of Water Resources, Division of Integrated Science and Engineering. 460 Glen Dr., Oroville, CA 95966.
Aidoo M., Bronx Community College/CUNY, Bronx, NY 10453
Colwell F., Oregon State University, College of Earth Ocean and Atmospheric Sciences; Oregon State University, Department of Microbiology. 226 Nash Hall, Corvallis, OR 97331

[*]Co-first authors
[+]Corresponding Author: suffridc@oregonstate.edu

SUPPLEMENTARY METHODS

Bioinformatics Analysis

PERMANOVA, significant differences between group variances, and environmental vectors were generated with the "adonis2", "betadisper" and "permutest", and "gg_envfit" commands, respectively. Constrained analysis of principal coordinates (CAP) was performed with the "ordinate" command from Phyloseq on bacterial and archaeal communites (not algal) with the variables, region, time point, and sample type, and was plotted with the "plot_ordination" command from Phyloseq. Richness and Shannon diversity index (ASV richness and evenness) values of whole communities (bacteria, archaea, and eukaryotic algae) for boxplots with the command "get_alphaindex" from MicrobiotaProcess. Kruskal Wallis tests on differences in Shannon diversity indices and on differences in dTRC concentrations between groups was performed with the command, "kruskal.test" from stats. Mantel tests were performed with the "mantel" command from vegan. Bray-Curtis dissimilarity values were calculated with the "distance" command from Phyloseq. Mantel tests were also performed between Bray-Curtis dissimilarity and Haversine distance by transforming latitude and longitude values of each sample site to Haversine distance values with the "distm" command from geospher. Mantel tests were performed on SW and HZ bacterial, archaeal, and algal communities separately. Spearman correlation coefficients were calculated with the function, "cor", p-values with the function, "cor.mtest", and correlograms plotted with "corrplot", all from corrplot.

SUPPLEMENTARY FIGURES AND TABLES

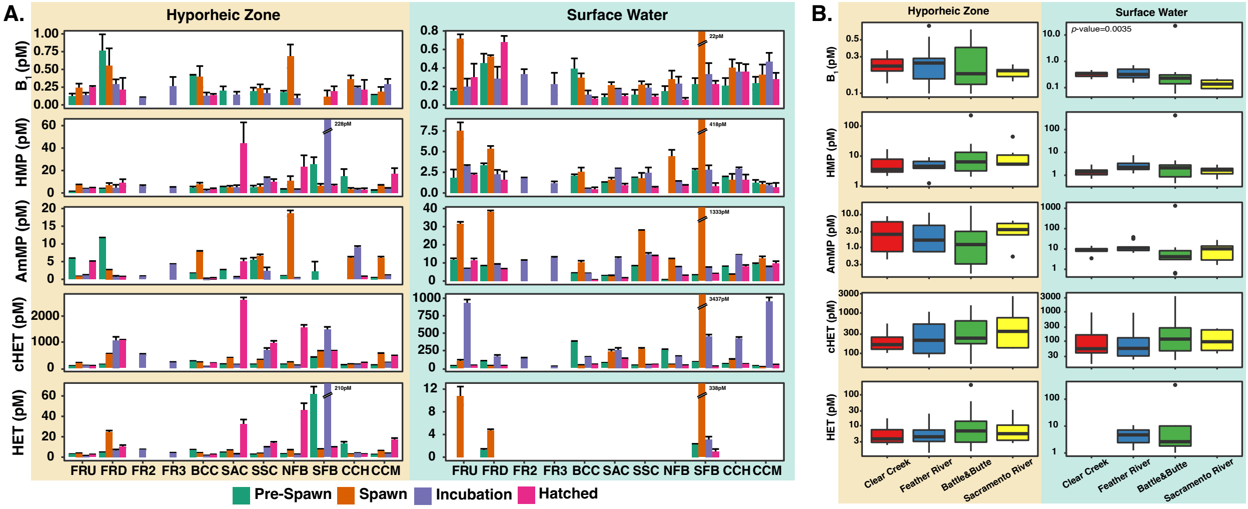

Supp. Figure 1: Regional variations in dTRC concentrations. *A. Concentrations of dTRCs in the river surface water (blue) and hyporheic zone (tan) at each sampling location and time point. Station codes are described in Methods. Bar height and error bars represent the mean and standard deviation of three technical replicates. No bars indicate that dTRCs were not detected in the samples, except for FR2 and FR3 where samples were only collected at the In-Gravel time point. Full dataset is in Table S1. B. Boxplots of hyporheic zone (tan) and surface water (blue) dTRC concentrations are binned by region. Colors indicate tributary: Feather River (Blue), Sacramento River (Yellow), Clear Creek (Red), Battle and Butte Creeks (Green). Kruskal-Wallis tests were used to test for significant differences in dTRC concentrations between regional bins; statistical significance is indicated where present.*

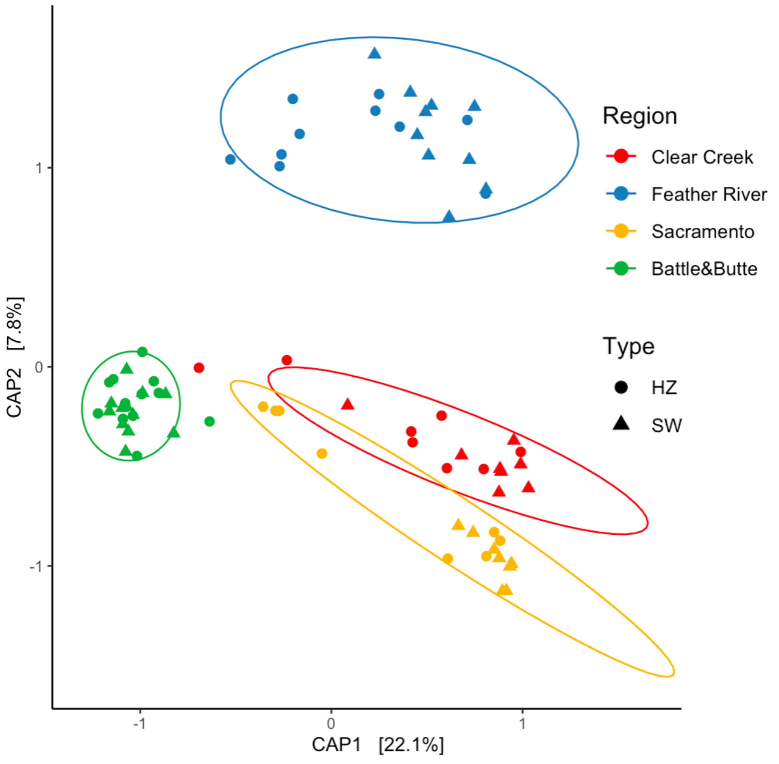

Supp. Figure 2: Constrained-analysis of principal coordinates displaying differences in bacterial, archaeal, and algal communities. *Data are constrained by the variables: region, time point, and sample type. CAP1 described 21.5% of variance and CAP2 described 8.3% of variance. Colors correspond to region and shapes correspond to sample type.*

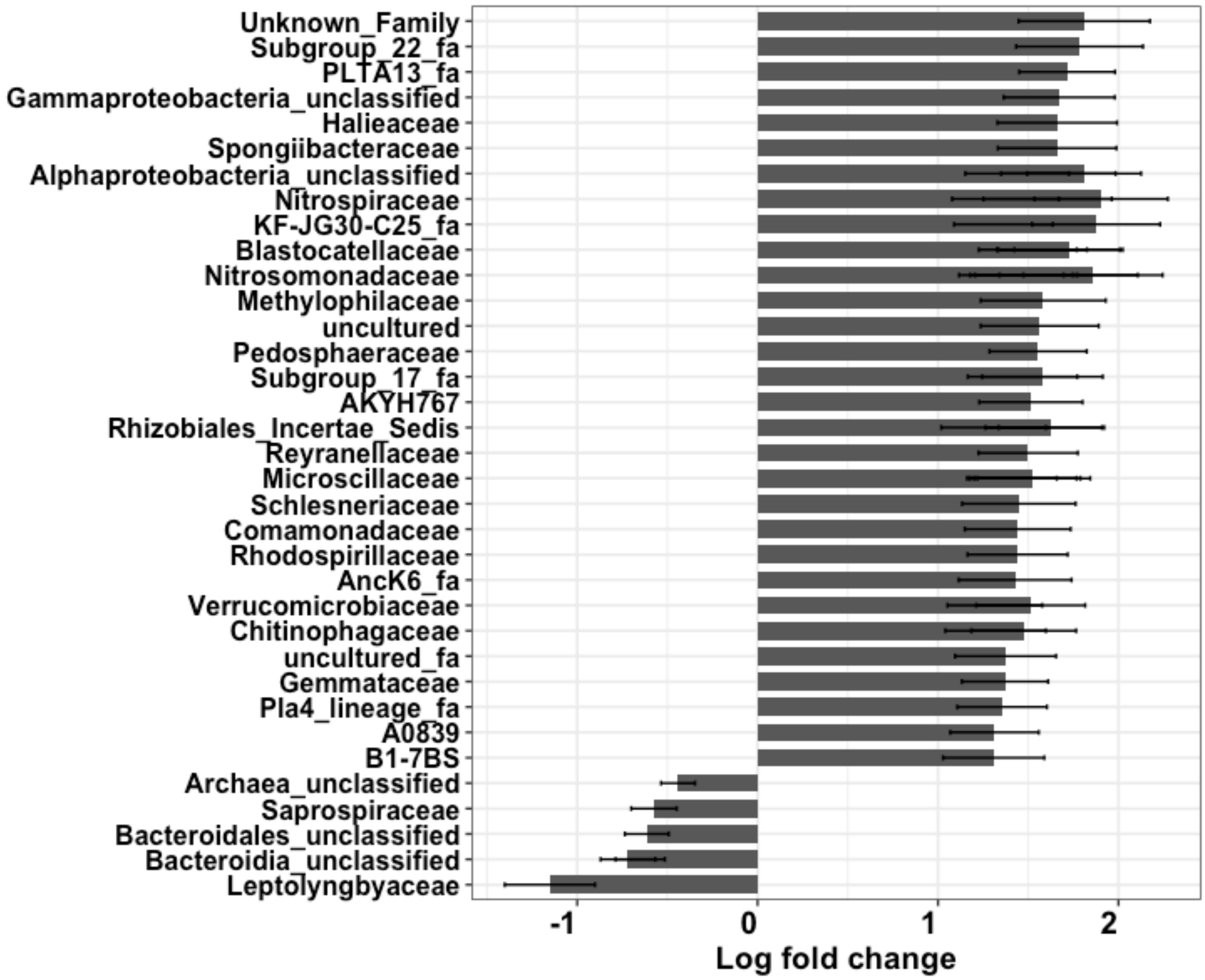

Supp. Figure 3: Families of ASVs that are differentially abundant in hyporheic zone samples (+log-fold change) compared to surface water samples (-log-fold change).

Supp. Table 1: dTRC concentrations. *Values are means and standard deviations of three technical replicates. All concentrations are in picomolar. Blanks indicate compound was undetectable in the sample.*

Time Point	Station	River	Type	B1	HMP	AmMP	cHET	HET
Pre-Spawn	FRU	Feather River	HZ	0.12 ± 0.04	1.24 ± 0.20	5.77 ± 0.21	78.67 ± 1.86	2.90 ± 0.15
Pre-Spawn	FRD	Feather River	HZ	0.76 ± 0.23	3.84 ± 0.20	11.50 ± 0.30	133.00 ± 4.36	4.63 ± 0.29
Pre-Spawn	BCC	Battle&Butte	HZ	0.41 ± 0.01	4.63 ± 1.14	1.63 ± 0.09	254.33 ± 16.92	7.28 ± 0.13
Pre-Spawn	SAC	Sacramento River	HZ	0.20 ± 0.06	5.12 ± 0.65	2.45 ± 0.29	139.33 ± 15.70	4.64 ± 0.14
Pre-Spawn	SSC	Sacramento River	HZ	0.20 ± 0.05	4.99 ± 1.62	5.51 ± 0.63	132.00 ± 10.58	2.74 ± 0.08
Pre-Spawn	NFB	Battle&Butte	HZ	0.18 ± 0.02	2.84 ± 0.76	0.91 ± 0.07	139.67 ± 3.51	2.97 ± 0.26
Pre-Spawn	SFB	Battle&Butte	HZ		25.67 ± 6.23	2.26 ± 2.75	387.00 ± 36.01	62.20 ± 6.99
Pre-Spawn	CCH	Clear Creek	HZ		14.93 ± 6.38		102.60 ± 23.40	13.60 ± 1.57
Pre-Spawn	CCM	Clear Creek	HZ	0.13 ± 0.01	2.17 ± 0.50	0.43 ± 0.03	103.33 ± 14.57	2.37 ± 0.27
Spawn	FRU	Feather River	HZ	0.24 ± 0.06	6.87 ± 0.73	0.76 ± 0.08	201.33 ± 5.86	3.75 ± 0.20
Spawn	FRD	Feather River	HZ	0.55 ± 0.25	6.99 ± 1.88	2.57 ± 0.17	538.33 ± 16.62	24.60 ± 1.45
Spawn	BCC	Battle&Butte	HZ	0.40 ± 0.15	7.83 ± 1.36	7.73 ± 0.31	223.33 ± 7.51	3.93 ± 0.37
Spawn	SAC	Sacramento River	HZ		5.27 ± 1.14		391.67 ± 8.39	6.54 ± 0.51
Spawn	SSC	Sacramento River	HZ	0.24 ± 0.05	5.46 ± 2.37	6.55 ± 0.51	314.00 ± 13.86	3.57 ± 0.18
Spawn	NFB	Battle&Butte	HZ	0.68 ± 0.17	11.04 ± 3.95	18.63 ± 0.81	223.33 ± 10.26	6.29 ± 1.07
Spawn	SFB	Battle&Butte	HZ	0.12 ± 0.09	6.43 ± 1.76		633.00 ± 33.05	7.65 ± 0.32
Spawn	CCH	Clear Creek	HZ	0.37 ± 0.05	3.29 ± 1.24	5.99 ± 0.41	139.67 ± 17.39	2.88 ± 0.19
Spawn	CCM	Clear Creek	HZ	0.19 ± 0.06	6.35 ± 0.91	6.06 ± 0.36	549.00 ± 27.73	6.06 ± 0.42
Incubation	FRU	Feather River	HZ	0.14 ± 0.04	3.76 ± 0.07	1.09 ± 0.19	90.90 ± 6.24	1.33 ± 0.22
Incubation	FRD	Feather River	HZ	0.30 ± 0.06	4.60 ± 2.72	0.73 ± 0.26	1061.33 ± 139.52	6.62 ± 0.95
Incubation	FR2	Feather River	HZ	0.10 ± 0.01	5.98 ± 1.00	0.73 ± 0.19	516.33 ± 29.67	7.47 ± 0.22
Incubation	FR3	Feather River	HZ	0.26 ± 0.13	4.54 ± 0.85	4.26 ± 0.08	221.67 ± 11.59	4.14 ± 0.27
Incubation	BCC	Battle&Butte	HZ	0.13 ± 0.04	2.05 ± 1.06	0.16 ± 0.13	54.73 ± 0.74	1.49 ± 0.23
Incubation	SAC	Sacramento River	HZ	0.14 ± 0.04	4.96 ± 2.01	0.53 ± 0.21	132.00 ± 4.36	2.84 ± 0.56
Incubation	SSC	Sacramento River	HZ	0.17 ± 0.06	12.97 ± 1.16	2.40 ± 0.97	705.33 ± 66.76	9.47 ± 0.74
Incubation	NFB	Battle&Butte	HZ	0.10 ± 0.05	2.88 ± 0.52	0.33 ± 0.09	99.73 ± 9.34	2.81 ± 0.12
Incubation	SFB	Battle&Butte	HZ		227.67 ± 116.84		1423.33 ± 185.02	209.67 ± 25.01
Incubation	CCH	Clear Creek	HZ	0.24 ± 0.02	2.95 ± 0.94	8.96 ± 0.44	135.67 ± 10.97	3.93 ± 0.18
Incubation	CCM	Clear Creek	HZ	0.29 ± 0.07	3.89 ± 0.65	1.05 ± 0.14	206.00 ± 8.00	3.55 ± 0.34
Hatched	FRU	Feather River	HZ	0.25 ± 0.01	4.04 ± 0.74	4.86 ± 0.30	86.97 ± 5.66	2.37 ± 0.37
Hatched	FRD	Feather River	HZ	0.22 ± 0.16	9.18 ± 3.08	0.71 ± 0.10	1076.67 ± 15.28	10.57 ± 1.28
Hatched	BCC	Battle&Butte	HZ	0.13 ± 0.02	3.31 ± 0.91	0.28 ± 0.17	187.33 ± 6.43	2.55 ± 0.22
Hatched	SAC	Sacramento River	HZ		44.37 ± 18.54	4.98 ± 0.88	2623.33 ± 95.04	32.63 ± 4.27
Hatched	SSC	Sacramento River	HZ		10.12 ± 2.23		973.00 ± 74.18	13.83 ± 1.22
Hatched	NFB	Battle&Butte	HZ		23.47 ± 10.09		1556.67 ± 105.99	46.23 ± 6.67
Hatched	SFB	Battle&Butte	HZ	0.19 ± 0.07	6.36 ± 1.19		657.67 ± 9.29	9.36 ± 0.76
Hatched	CCH	Clear Creek	HZ	0.21 ± 0.14	2.85 ± 1.38	0.67 ± 0.20	194.67 ± 19.63	2.87 ± 0.27
Hatched	CCM	Clear Creek	HZ		16.97 ± 5.14		465.33 ± 19.43	17.40 ± 1.32
Pre-Spawn	FRU	Feather River	SW	0.15 ± 0.02	1.87 ± 0.97	11.83 ± 0.71	29.40 ± 3.56	
Pre-Spawn	FRD	Feather River	SW	0.45 ± 0.10	3.36 ± 0.23	8.25 ± 0.25	96.53 ± 7.34	1.26 ± 0.21
Pre-Spawn	BCC	Battle&Butte	SW	0.39 ± 0.11	2.11 ± 0.16	4.20 ± 0.26	371.67 ± 15.50	
Pre-Spawn	SAC	Sacramento River	SW	0.08 ± 0.03	1.23 ± 0.09	2.91 ± 0.02	70.57 ± 4.68	
Pre-Spawn	SSC	Sacramento River	SW	0.11 ± 0.05	1.86 ± 0.03	8.25 ± 0.21	37.97 ± 1.66	
Pre-Spawn	NFB	Battle&Butte	SW	0.15 ± 0.05		0.67 ± 0.03	259.67 ± 10.07	
Pre-Spawn	SFB	Battle&Butte	SW	0.23 ± 0.06	2.77 ± 0.16	3.05 ± 0.26	88.50 ± 0.95	2.21 ± 0.13
Pre-Spawn	CCH	Clear Creek	SW	0.21 ± 0.08	1.90 ± 0.10	7.76 ± 0.39	55.13 ± 6.07	
Pre-Spawn	CCM	Clear Creek	SW	0.23 ± 0.07	1.23 ± 0.36	9.31 ± 0.53	29.13 ± 2.42	
Spawn	FRU	Feather River	SW	0.72 ± 0.05	7.54 ± 1.00	31.73 ± 0.93	103.27 ± 15.67	10.84 ± 1.61
Spawn	FRD	Feather River	SW	0.52 ± 0.02	5.38 ± 0.30	38.23 ± 0.71	31.07 ± 4.10	4.72 ± 0.21
Spawn	BCC	Battle&Butte	SW	0.30 ± 0.04	2.56 ± 0.51	10.39 ± 0.81	37.83 ± 7.00	
Spawn	SAC	Sacramento River	SW	0.21 ± 0.03	1.62 ± 0.23	2.75 ± 0.43	237.33 ± 26.01	
Spawn	SSC	Sacramento River	SW	0.22 ± 0.04	1.76 ± 0.67	27.80 ± 0.35	272.67 ± 16.29	
Spawn	NFB	Battle&Butte	SW	0.28 ± 0.09	4.47 ± 0.75	12.03 ± 0.42	49.63 ± 2.66	
Spawn	SFB	Battle&Butte	SW	22.30 ± 12.51	418.33 ± 49.89	1333.33 ± 102.63	3436.67 ± 94.52	338.33 ± 40.81
Spawn	CCH	Clear Creek	SW	0.41 ± 0.09	1.60 ± 0.72	3.48 ± 0.35	119.00 ± 11.53	
Spawn	CCM	Clear Creek	SW	0.33 ± 0.11	1.09 ± 0.27	12.50 ± 1.13	16.67 ± 2.38	
Incubation	FRU	Feather River	SW	0.20 ± 0.09	3.26 ± 0.12	6.77 ± 0.20	932.33 ± 49.33	
Incubation	FRD	Feather River	SW	0.28 ± 0.13	2.26 ± 0.53	8.98 ± 0.47	166.67 ± 20.82	
Incubation	FR2	Feather River	SW	0.33 ± 0.05	1.77 ± 0.10	11.10 ± 0.50	140.33 ± 9.29	
Incubation	FR3	Feather River	SW	0.23 ± 0.12	1.21 ± 0.19	12.77 ± 0.61	23.57 ± 1.55	
Incubation	BCC	Battle&Butte	SW	0.11 ± 0.04	0.52 ± 0.04	4.16 ± 0.12	156.33 ± 4.73	
Incubation	SAC	Sacramento River	SW	0.17 ± 0.01	2.91 ± 0.07	12.53 ± 0.70	258.67 ± 30.86	
Incubation	SSC	Sacramento River	SW	0.19 ± 0.04	2.48 ± 0.60	14.80 ± 0.75	44.47 ± 4.57	
Incubation	NFB	Battle&Butte	SW	0.23 ± 0.07	1.39 ± 0.06	7.60 ± 0.37	166.33 ± 5.69	
Incubation	SFB	Battle&Butte	SW	0.33 ± 0.12	2.81 ± 0.53	7.25 ± 0.44	456.00 ± 23.07	3.14 ± 0.48
Incubation	CCH	Clear Creek	SW	0.36 ± 0.09	2.94 ± 0.16	14.37 ± 0.12	426.67 ± 18.15	
Incubation	CCM	Clear Creek	SW	0.47 ± 0.10	0.96 ± 0.19	7.70 ± 0.42	952.67 ± 59.41	
Hatched	FRU	Feather River	SW	0.30 ± 0.15	2.20 ± 0.16	11.40 ± 1.08	33.30 ± 6.10	
Hatched	FRD	Feather River	SW	0.68 ± 0.07	1.63 ± 0.97	6.45 ± 0.38	31.87 ± 3.01	
Hatched	BCC	Battle&Butte	SW	0.07 ± 0.02	0.45 ± 0.24	0.61 ± 0.02	52.63 ± 0.50	
Hatched	SAC	Sacramento River	SW	0.09 ± 0.02	0.96 ± 0.17	1.63 ± 0.15	129.33 ± 12.06	
Hatched	SSC	Sacramento River	SW	0.09 ± 0.02	0.66 ± 0.10	13.60 ± 0.52	49.57 ± 2.25	
Hatched	NFB	Battle&Butte	SW	0.06 ± 0.02	0.87 ± 0.12	2.98 ± 0.20	38.97 ± 3.20	
Hatched	SFB	Battle&Butte	SW	0.22 ± 0.04	0.84 ± 0.37	3.84 ± 0.32	22.70 ± 4.00	1.02 ± 0.41
Hatched	CCH	Clear Creek	SW	0.36 ± 0.08	1.61 ± 0.62	8.17 ± 0.70	54.97 ± 4.97	
Hatched	CCM	Clear Creek	SW	0.28 ± 0.07	0.70 ± 0.52	9.70 ± 1.25	44.13 ± 7.12	
